# Radical climate protests shaped portrayals of moderate activists and reader attitudes in German news media

**DOI:** 10.1038/s44168-026-00361-7

**Published:** 2026-07-27

**Authors:** Lukas Mayrhofer, Simon Fassnacht, Markus Foramitti, Jana K. Köhler, Boryana Todorova, Claus Lamm, Mauricio Martins

**Affiliations:** 1 Social, Cognitive and Affective Neuroscience Unit, Faculty of Psychology, University of Vienna, Vienna, Austria; 2 Max Planck Institute for Biological Cybernetics, Tübingen, Germany; 3 Department of Clinical and Health Psychology, Faculty of Psychology, University of Vienna, Vienna, Austria; 4 Environmental Psychology, Department of Cognition, Emotion, and Methods in Psychology, Faculty of Psychology, University of Vienna, Vienna, Austria; 5 CICANT, ECATI, Lusófona University, Lisbon, Portugal

**Keywords:** Environmental social sciences, Psychology, Psychology

## Abstract

Radical climate protests have been theorized to shape perceptions of moderate climate groups, a dynamic known as the radical flank effect (RFE). We examine this phenomenon through a longitudinal analysis of German media discourse on climate protests. Using validated large language model prompts, we annotated stances and anger in 2376 news articles and 225,121 user comments covering the moderate activist group Fridays for Future (FFF) and the more radical Last Generation (LG). Short-term increases in LG protest coverage were associated with increased support for FFF in news articles and left-leaning outlets’ user comments, but with decreased support in right-leaning comments. However, these patterns were not observed over longer time periods. Although anger expressed in the general coverage of climate protests impacted subsequent support for FFF, this effect was not specific to coverage of radical protests. These findings highlight the temporally bounded and ideologically contingent nature of RFEs in media discourse.

## Introduction

In recent years, climate activism has increasingly relied on public protests to demand political action on climate change^1^. One prominent example is Fridays for Future (FFF), which mobilized millions worldwide and successfully elevated climate change on political agendas^2,3^. Yet despite this early momentum, the impact of such protests on emission trends remains uncertain^4^. In response to this uncertainty, newer groups, such as Just Stop Oil and Last Generation (LG) have adopted more radical protest tactics that directly interfere with everyday activities^5^, and spurred public debate over whether such tactics ultimately advance or undermine the goals of the climate movement. These developments have sharpened a central tension facing social movements: actions that attract attention and urgency may simultaneously provoke backlash and erode public support^6,7^.

Empirical research on the effects of disruptive climate protests paints a mixed picture. Some studies report that disruptive protests reduce public support for activists^8^, identification with the movement^6,9^, or endorsement of proposed climate policies^7,10^. Other work finds no such negative effects^11,12^, or even positive outcomes on pro-environmental attitudes^7,13^, concern^14^, and media attention for climate issues^15,16^. Notably, these contrasting findings often emerge from different methodological contexts. Studies reporting negative effects have largely relied on laboratory experiments using fictional activist groups^6–10^, whereas studies observing neutral or positive effects more often examine real activist groups or naturalistic field settings^12–14^. As a result, the existing literature does not converge on whether disruptive climate protests systematically harm or help public support.

One reason for this lack of convergence may be that existing approaches capture different stages of public response. Laboratory experiments typically present participants with unfamiliar activist groups or protest actions, thereby measuring immediate, first-exposure reactions that may be dominated by annoyance, threat, or reactance. In contrast, field studies observe populations with heterogeneous and often repeated exposure to climate activists over extended periods. Under such conditions, initial negative reactions may attenuate through habituation, normalization, or selective disengagement, yielding more neutral or even positive aggregate responses over time. Importantly, this does not imply that disruptive protests are evaluated positively from the outset; rather, their effects may change as exposure accumulates. Current research designs are poorly equipped to distinguish between short- and long-term dynamics, underscoring the need for diachronic analyses on the perception of climate activism in real-world settings.

For most citizens, climate protests are not experienced directly but through news media coverage. Media portrayals, therefore, play a central role in shaping how climate activism is perceived, and what effects it has^17–19^. Prior research shows that protest coverage is often framed negatively, even for non-disruptive actions, and that this framing varies systematically with news outlets’ political orientation^20,21^. In Germany, for example, right-leaning outlets tend to depict climate activists in more criminalizing and emotionally charged terms than left-leaning outlets, which more often emphasize protesters’ demands and motivations^21^. These patterns are especially pronounced for disruptive protests, where negative framing intensifies and ideological differences between outlets narrow^21,22^. Despite this extensive literature on media framing, existing work has largely examined how individual protest events or activist groups are portrayed in isolation, leaving open the question of how coverage of one group may shape the portrayal of another pursuing similar goals.

This relational gap is central to theories of the radical flank effect (RFE), which describe how the presence of a more radical faction within a social movement influences public support for more moderate factions^23^. Radical groups may undermine support for moderates by contaminating them with negative associations (negative RFE) or increase support for moderates by making them appear more reasonable by comparison (positive RFE)^24^. While RFEs have been documented in historical case studies^24,25^, evidence remains mixed in the context of climate activism^9,10,26–28^. Moreover, most existing studies examine RFEs at a single point in time or under controlled conditions, providing limited insight into how these effects unfold in longitudinal, real-world media environments and how media portrayals affect readers’ perceptions of climate activists.

Beyond cognitive evaluations, RFEs may also operate through emotional responses toward activist groups. Emotional reactions toward social groups are central drivers of intergroup attitudes and behavior, including support for collective action and social movements^29,30^. Among these emotions, anger is particularly relevant to climate activism. Unlike emotions, such as sadness or anxiety, which are often associated with withdrawal, helplessness, or paralysis^31,32^, anger is characterized by strong action tendencies and has been linked to collective action^33–35^ and pro-environmental intentions^36,37^. Media coverage of climate protests frequently evokes anger, especially when protests are portrayed as norm-violating or disruptive^21^, making anger a plausible affective pathway through which radical activism could influence evaluations of more moderate groups. At the same time, emotions play a nuanced role in intergroup processes, and different emotions may be directed at different targets, such as activists, political institutions, or climate change itself. Here, we focus on anger as a theoretically central and empirically tractable case, while recognizing that other emotions, such as hope or anxiety may also shape RFEs and warrant future investigation^38^.

In the present study, we examine RFEs in a real-world media environment by analyzing news articles and user comments from eight major German news outlets: four left-leaning and four right-leaning. We quantify evaluative stances toward the activist group FFF and anger expression in protest-related news articles using natural language processing methods validated against human coding. This design allows us to examine how portrayals of a moderate activist group (FFF) change over time, across political contexts, and in relation to coverage of a more radical group (LG), within institutional media and user responses.

Throughout this article, we use the terms *radical* and *moderate* to distinguish activist groups based on their predominant protest strategies. In the German context, FFF and LG have been consistently portrayed and perceived as occupying distinct positions along this dimension. Multiple studies show that German news media frame LG as more polarizing, confrontational, and violent than FFF, and use substantially more negative language when reporting on LG^5,20,22^. Public opinion data further indicate that LG is evaluated more negatively than FFF in representative samples^30^. These differences reflect the groups’ protest strategies: FFF primarily organized publicly announced and authorized demonstrations, whereas LG mainly engaged in unannounced traffic blockades and acts of vandalism^5,22^. Consistent with both empirical evidence and radical flank theory, which conceptualizes interactions among factions within a movement, we treat FFF as the moderate faction and LG as the radical faction in our analyses. We acknowledge that individual protest events may deviate from these general tendencies, but the distinction reflects predominant patterns of portrayal and perception during the period under study.

Identifying RFEs in observational media data requires a relational rather than purely temporal interpretation of change. Public stances toward FFF evolved over time for many reasons independent of radical protest, including continued non-disruptive demonstrations, rising climate awareness, and broader societal developments. Consequently, changes in FFF stances that coincide with LG coverage cannot be interpreted in isolation as effects of radical activism. We therefore define RFEs counterfactually as deviations from a baseline trajectory reflecting how stances toward FFF would be expected to change in the absence of LG’s more radical protests. In our design, media coverage of FFF itself serves as this baseline, capturing ongoing, less radical activism and general time-varying influences. A positive RFE occurs when coverage of LG predicts more favorable stances toward FFF than would be expected based on coverage of FFF itself, whereas a negative RFE occurs when LG coverage predicts less favorable stances than this baseline. Conceptually, RFEs therefore reflect the *difference* between the effect of radical-group coverage and the effect of continued moderate-group coverage on evaluations of the moderates. Thus, absolute increases or decreases in FFF support over time do not, by themselves, constitute evidence for an RFE unless it diverges from this baseline counterfactual comparison.

Based on this framework, we tested whether stances toward FFF became more favorable following the emergence of LG (H1) and whether RFEs were predicted by the cumulative volume of LG coverage over longer time windows (H2.1) and by short-term fluctuations in LG coverage (H2.2). Finally, we hypothesized that higher levels of anger expressed in recent coverage of LG protests would predict more favorable stances toward FFF than the baseline (H3). These hypotheses were pre-registered (https://osf.io/f5gza).

## Results

### Results overview and analytical approach

Table 1 provides an overview of all hypotheses and observed effects, serving as a roadmap for the Results section. For each hypothesis, the table summarizes (i) the direction of change in stances toward FFF over time and (ii) whether this change constitutes an RFE, defined as a deviation from the counterfactual baseline implied by FFF coverage itself. Throughout the Results, *stances* are measured on an ordinal five-point scale ranging from “strongly against” to “strongly in favor” of FFF. Importantly, our analyses focus on dynamic temporal trends rather than static mean differences: effects reflect changes in the trajectory of these ordinal stances over time and how those trajectories differ following coverage of LG relative to the FFF coverage baseline.

**Table 1:** Summary of hypotheses and observed radical flank effects (RFE)

Hypothesis	Source	Leaning	FFF support (raw)	LG v FFF contrast (RFE*)
**H1: LG emergence**	Articles	L	n.s.	=
		R	↑ after LG (trend)	> ( + RFE, trend)
	Comments	L	n.s.	=
		R	n.s.	=
**H2.1: Long-term LG coverage (cummulative #articles)**	Articles	L	n.s.	=
		R	n.s.	=
	Comments	L	↑	< (-RFE)
		R	↓	=
**H2.2: Short-term LG coverage (#articles within 30-day window)**	Articles	L	n.s.	> ( + RFE)
		R	n.s.	> ( + RFE)
	Comments	L	↑	> ( + RFE)
		R	↓	< (-RFE)
**H3: Anger in LG coverage (within 30-day window)**	Articles	L	↓ when anger rises	=
		R	↓ when anger rises	=
	Comments	L	↑ when anger rises	=
		R	↑ when anger rises	=

*An RFE is inferred only when the effect of LG coverage differs significantly from the baseline implied by FFF coverage.

RFE refers to the radical flank effect, defined as the influence of a more radical group on stances toward a more moderate group. In this study, Last Generation (LG) represents the radical faction and Fridays for Future (FFF) the moderate faction. FFF support (raw): direction of change in stances toward FFF after LG coverage but without baseline contrast; RFE (LG v FFF): defined counterfactually as the difference between the effect of LG coverage on FFF stances and the baseline effect implied by FFF coverage itself. Symbols denote the direction of this difference: > (+RFE) indicates that LG coverage predicts more favorable stances toward FFF than FFF coverage, and a positive RFE; < (−RFE) indicates that LG coverage predicts less favorable stances and a negative RFE; = indicates no differential effect (no RFE); n.s.: non-significant effects (*p* > 0.05) prior to Bonferroni–Holm correction for multiple testing; trend: significant effects (*p* < 0.05) that did not survive the Bonferroni–Holm correction for multiple testing; Leaning: refers to the political orientation of news outlets: (L)eft and (R)ight.

All statistical models were specified as preregistered (https://osf.io/f5gza). In line with the preregistered analytical strategy and to facilitate interpretability, non-significant higher-order interaction terms were removed stepwise while retaining all lower-order terms. A complete overview of preregistered and final model specifications for each hypothesis (including where higher-order interactions were retained or removed) is provided in Supplementary Table S2. Full model equations, results, and assumption checks are reported in the Supplementary Materials A–C.

The following sections unpack these results in detail, organized by hypothesis, and examine how the temporal dynamics of stances towards FFF vary by news outlets’ political orientation (left vs. right) and by content source (news articles vs. user comments).

### Validation of GPT-based annotations

Before testing our hypotheses, we assessed the reliability of GPT-4-Turbo for annotating stances toward FFF and expressions of anger in news articles (Box S1) and user comments (Box S2). GPT annotations were compared with majority ratings from three independent human coders—defined as the category selected by at least two of the three raters—on randomly selected subsets of the data (Box S3; see Methods: Language processing and model validation for details).

For stance annotations, GPT-4-Turbo showed strong agreement with human ratings in both news articles (Kendall’s W = 0.81, ICC = 0.68, *p* < 0.001) and user comments (W = 0.91, ICC = 0.86, κ = 0.64, all *p* < 0.001). Agreement between GPT and the human majority rating was comparable to, and in several cases higher than, agreement among the human raters themselves.

For anger annotations, agreement between GPT and human ratings was again substantial for both news articles (W = 0.77, ICC = 0.57, *p* < 0.001) and user comments (W = 0.76, ICC = 0.52, *p* < 0.001), and exceeded human inter-rater agreement.

Together, these results indicate that GPT-4-Turbo provides reliable and conservative annotations of both stances and anger in our corpus, supporting its use in subsequent analyses.

### LG emergence did not produce an RFE but widened ideological divergence in media coverage (H1)

**Fig. 1:**
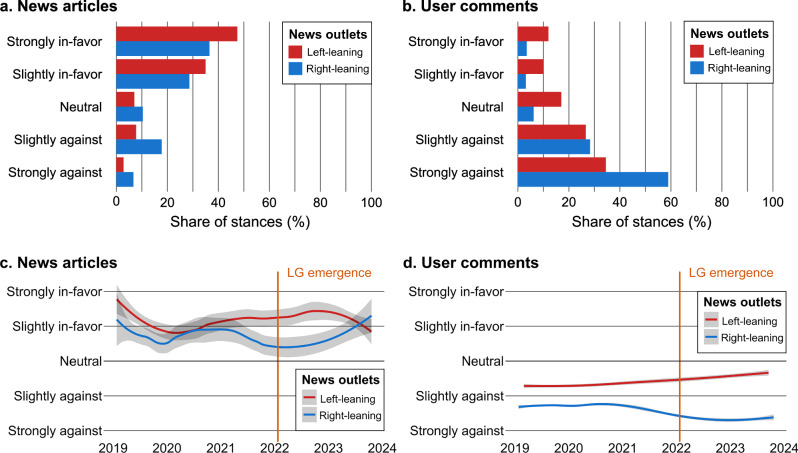
Distribution of stances towards FFF annotated by GPT4-Turbo. Distribution of stances towards FFF in **a** news articles and **b** user comments. Diachronic change in mean stances and their respective 95% confidence intervals in **c** news articles and **d** user comments. The orange vertical line indicates the beginning of disruptive LG protests.

Figure 1 summarizes the distribution and temporal development of stances toward FFF in news articles and user comments (Table S3). Overall, most news articles were favorable toward FFF in both right-leaning (65%) and left-leaning outlets (82%). In contrast, user comments were predominantly unfavorable, especially in right-leaning outlets (88% unfavorable vs. 60% in left-leaning outlets).

We tested whether the emergence of LG was associated with a shift in *trends* (i.e., stance slopes over time) toward FFF (H1). We fitted a proportional-odds (PO) model with the three-way interaction LG emergence (pre/post) × outlet’s political orientation (left/right) × time (months since the emergence of FFF; see Supplementary Materials A1). Because our design defines RFEs as changes in trends, the key question is whether stance slopes toward FFF changed after LG’s emergence *within outlets of the same political orientation*, not merely whether left- and right-leaning outlets differed.

### News articles

**Fig. 2:**
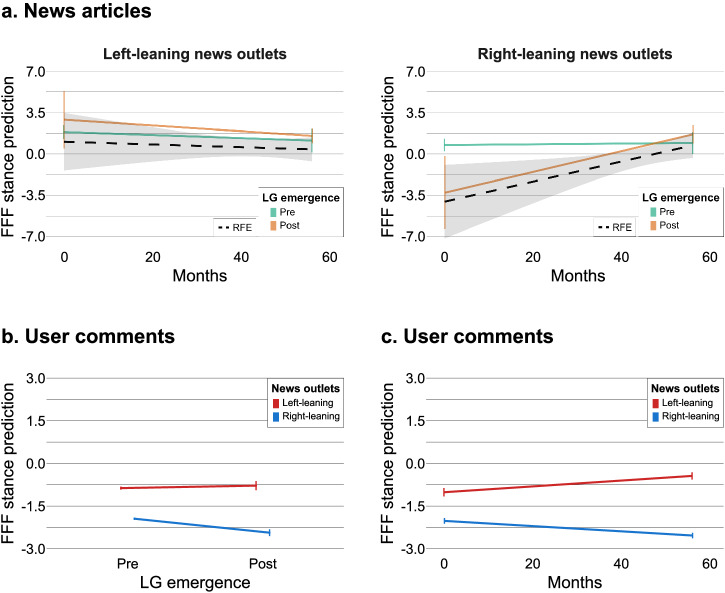
Effects of the emergence of Last Generation (LG) on stances toward Fridays for Future (FFF) (H1). Predicted marginal effects (±SE) from proportional-odds models testing changes in temporal trends of FFF stances following the emergence of LG. The dashed line denotes the counterfactual radical flank effect (RFE), defined as the marginal difference in stance trajectories between the pre- and post LG condition. **a** News articles. A significant three-way interaction between LG emergence (pre vs. post), outlet political orientation (left vs. right), and time indicates increasing divergence in stance slopes after LG emerged: stances toward FFF became more favorable in right-leaning outlets after LG protests emerged, but did not change in left-leaning outlets. However, within-orientation pre–post comparisons were not significant after Bonferroni-Holm correction, indicating no robust RFE. **b** User comments. A significant interaction between LG emergence and political orientation reflects a decline in FFF support in right-leaning outlets after LG emerged, with no corresponding change in left-leaning outlets. **c** User comments. A significant interaction between political orientation and time shows increasing ideological divergence over time, with stances becoming more favorable in left-leaning comments and less favorable in right-leaning comments. No interaction between LG emergence and time was observed, indicating no RFE in comments.

In news articles, the three-way interaction was significant (OR = 1.10, SE = 1.04, *p* = 0.03; Table S4), indicating that left-right differences in stance trends changed after LG’s emergence. Post-hoc comparisons showed that this effect reflected greater divergence in stance slopes between left- and right-leaning outlets after LG emerged (Δβ = −0.11, SE = 0.04, *p* = 0.03; Fig. 2a): right-leaning outlets’ support increased over time, whereas left-leaning outlets’ support declined.

However, when testing the *pre-post* change in stance slopes within each political orientation, we found no reliable shift: left-leaning outlets showed no change (Δβ = 0.01, SE = 0.03, *p* = 0.85), and right-leaning outlets showed only a marginal trend of rising support (Δβ = 0.09, SE = 0.03, *p* = 0.05) that did not survive Holm–Bonferroni correction. Thus, although ideological divergence increased after LG’s emergence, we did not observe a robust shift from the pre-LG baseline within either political camp. Therefore, H1 did not provide evidence for a RFE in news articles.

### User comments

In user comments, the corresponding three-way interaction was not significant (OR = 0.99, SE = 1.01, *p* = 0.40; Table S4), providing no evidence that LG’s emergence altered stance trajectories toward FFF. After removing non-significant higher-order interactions, the simplified model retained two effects (Fig. 2b, c). First, there was an LG emergence × political orientation interaction (OR = 0.56, SE = 1.13, *p* < 0.001), driven by a drop in support in the right-leaning outlet following LG’s emergence (Δβ = −0.49, SE = 0.07, *p* < 0.001) but no change in the left-leaning outlet (Δβ = 0.09, SE = 0.10, *p* = 0.37). Second, there was a political orientation × time interaction (OR = 0.98, SE = 1.00, *p* < 0.001), indicating that comment stances diverged over time (Δβ = 0.02, SE = 0.003, *p* < 0.001), becoming more supportive in left-leaning outlets and less supportive in right-leaning outlets. Crucially, the LG emergence × time interaction that would represent a RFE remained non-significant (OR = 1.01, SE = 1.00, *p* = 0.10) and was removed from the model.

In sum, H1 provides no robust evidence that LG’s emergence increased support for FFF relative to baseline trends. Instead, LG’s emergence coincided with greater divergence in support between left and right across both news articles and comments (Table 1; Fig. 2).

### Long-term protest coverage did not yield a robust RFE, except for a negative effect in left-leaning user comments (H2.1)

We tested whether long-term exposure to LG protests predicted changes in the trajectory of stances toward FFF over time (H2.1). We fitted a PO model with a three-way interaction between the cumulative number of protest-related articles since the emergence of FFF, outlet political orientation (left vs. right), and protest type (i.e., counterfactual condition; LG vs. FFF), with time (months since the emergence of FFF) included to model temporal trends (see Supplementary Materials A2). Although cumulative coverage and time were highly correlated (r = 0.78), excluding time did not substantively change the pattern of results.

Because our design defines RFEs as changes in slopes rather than mean differences, the key question is whether cumulative LG coverage altered the trajectory of FFF support relative to the counterfactual baseline implied by cumulative FFF coverage.

### News articles

For news articles, we did not observe a significant three-way interaction between cumulative coverage, political orientation, and protest type (OR = 0.97, SE = 1.06, *p* = 0.59; Table S5), indicating no differential long-term effect of LG coverage relative to the counterfactual FFF baseline. After removal of non-significant higher-order terms (Table S2), a significant interaction remained between cumulative coverage and political orientation (OR = 1.06, SE = 1.03, *p* = 0.02).

**Fig. 3:**
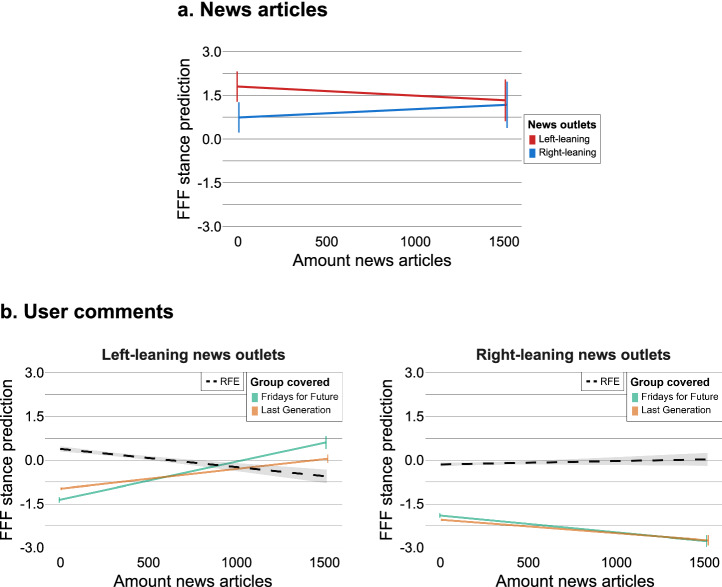
Effects of cumulative protest coverage on stances toward Fridays for Future (H2.1). Predicted marginal effects ( ± SE) from post-hoc comparisons of significant interactions in models testing H2.1. The dashed line indicates the estimated radical flank effect (RFE), defined as the deviation of LG coverage effects from the baseline trajectory implied by FFF coverage. **a** News articles. Increasing cumulative coverage of climate protests was associated with more favorable stances toward FFF in right-leaning outlets relative to left-leaning outlets, with no differential effect between LG and FFF coverage. **b** User comments. A significant three-way interaction between cumulative coverage, outlet political orientation, and protest type revealed that, in left-leaning outlets, cumulative LG coverage was associated with less favorable stances toward FFF than the FFF baseline.

Post-hoc analyses showed that as cumulative protest coverage increased, stances toward FFF became more favorable in right-leaning outlets and less favorable in left-leaning outlets (Δβ = −0.06, SE = 0.03, *p* = 0.02; Fig. 3a), mirroring results from H1. Importantly, this pattern did not differ between LG and FFF coverage. Thus, while cumulative protest reporting was associated with diverging trajectories of support towards FFF across political orientations, these effects were not specific to LG coverage and therefore do not constitute evidence for a RFE in news articles.

### User comments

In contrast, user comments showed a significant three-way interaction between cumulative coverage, political orientation, and protest type (OR = 1.07, SE = 1.01, *p* < 0.001; Table S5), indicating that the long-term effect of protest coverage on FFF stances differed between LG and the baseline condition.

Post-hoc analyses revealed that, in left-leaning outlets, cumulative coverage was associated with greater overall support for FFF; however, this increase was significantly weaker following LG coverage than following FFF (baseline) coverage. Specifically, the odds of more favorable stances toward FFF increased by 14% per 100 FFF-related articles, but by only 7% per 100 LG-related articles (Δβ = 0.06, SE = 0.01, *p* < 0.001; Fig. 3b). In right-leaning outlets, cumulative coverage predicted declining support for FFF, but this effect did not differ between LG and FFF coverage (Δβ = 0.01, SE = 0.01, *p* = 0.19).

Taken together, these results indicate a negative long-term RFE in left-leaning user comments, where LG coverage dampened the otherwise positive trajectory of FFF support relative to the baseline. No long-term RFE was observed in right-leaning comments or in news articles.

### Short-term LG coverage produces strong but ideologically asymmetric RFEs (H2.2)

We tested whether short-term exposure to LG protests predicted changes in the trajectory of stances toward FFF (H2.2). We fitted a PO model with a three-way interaction between the number of recent articles covering climate protests within a 30-day window, protest type (LG vs. FFF), and outlet political orientation (left vs. right), with time included to account for long-term temporal trends (see Supplementary Materials A3). The critical test was whether short-term LG coverage altered the slope of FFF support relative to the counterfactual baseline.

### News articles

In news articles, the three-way interaction between recent coverage, protest type, and political orientation did not reach significance (OR = 1.20, SE = 1.12, *p* = 0.09; Table S6). After model simplification (Table S2), a significant interaction between recent coverage and protest type emerged (OR = 1.20, SE = 1.06, *p* < 0.01). For every ten additional articles published in the prior 30 days, the odds of more favorable stances toward FFF were 20% higher following LG coverage compared to baseline FFF coverage.

**Fig. 4:**
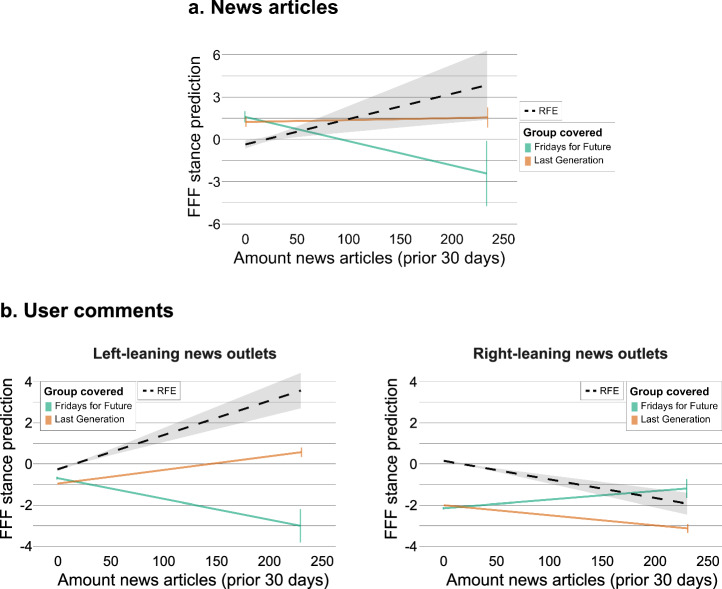
Effects of short-term protest coverage on stances toward Fridays for Future (H2.2). Predicted marginal effects ( ± SE) from post-hoc comparisons of significant interactions in models testing H2.2. The dashed line indicates the estimated radical flank effect (RFE), defined as the difference of LG coverage effects from the baseline trajectory implied by FFF coverage. **a** News articles. Increasing FFF coverage in the prior 30 days was associated with declining support for FFF, whereas increasing LG coverage was associated with more favorable trajectories, consistent with a positive RFE. **b** User comments. A significant three-way interaction between recent coverage, outlet political orientation, and protest type revealed opposing RFEs: in left-leaning outlets, LG coverage predicted increasing support for FFF relative to the FFF baseline, whereas in right-leaning outlets, LG coverage predicted declining support.

Post-hoc comparisons showed that this effect was driven by a decline in FFF support as recent FFF coverage increased, whereas support remained stable as LG coverage increased (Δβ = −0.18, SE = 0.06, *p* < 0.01; Fig. 4a). Thus, recent LG coverage predicted more favorable FFF trajectories than the FFF baseline, consistent with a positive RFE. Notably, this pattern reflects a short-term “fatigue” effect of repeated FFF coverage rather than a direct increase in support following LG coverage.

### User comments

In user comments, we observed a significant three-way interaction between recent coverage, protest type, and political orientation (OR = 0.77, SE = 1.02, *p* < 0.001; Table S6). Post-hoc analyses revealed a marked ideological asymmetry (Fig. 4b). In left-leaning outlets, the odds of more favorable FFF stances increased by 7% for every 10 LG articles but decreased by 10% for every 10 FFF articles (Δβ = 0.17, SE = 0.02, *p* < 0.001), indicating a positive RFE. In right-leaning outlets, this pattern reversed: FFF coverage predicted modest increases in support (4%), whereas LG coverage predicted decreases (5%) for every ten articles (Δβ = 0.09, SE = 0.01, *p* < 0.001), corresponding to a negative RFE.

Taken together, these findings show that short-term LG coverage reliably predicts RFEs. In news articles, this effect was consistent across political orientations. In user comments, however, its direction depended on audience ideology: LG coverage increased relative support for FFF in left-leaning outlets but decreased it in right-leaning outlets.

### Anger varies across media contexts but does not mediate RFEs (H3)

**Fig. 5:**
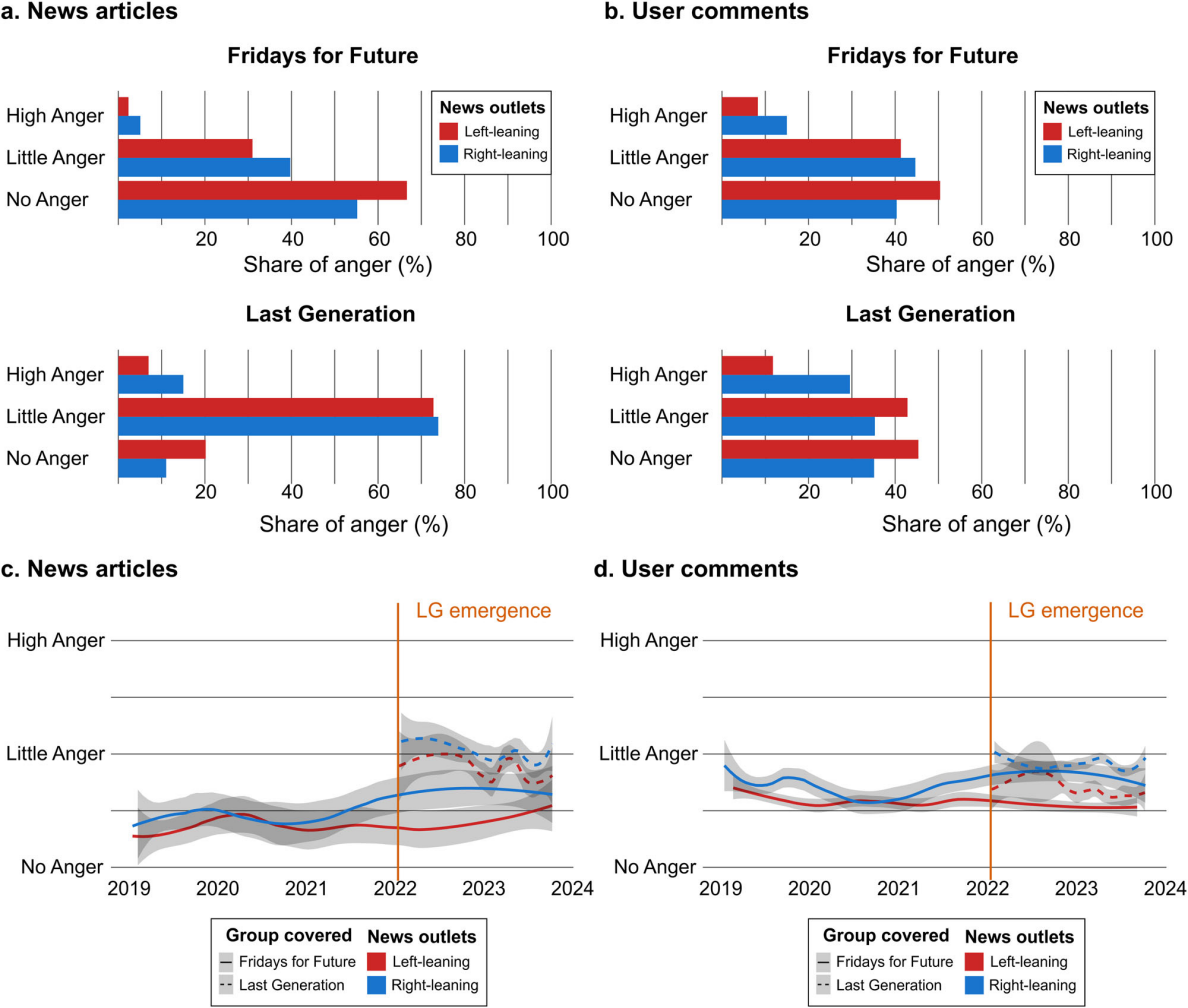
Distribution and temporal dynamics of anger in protest-related coverage. Share of anger levels in **a** news articles and **b** user comments. **c**, **d** Show smoothed mean anger levels in protest-related news coverage over the prior 30 days with 95% confidence intervals, for articles and comments, respectively. The vertical line indicates the onset of disruptive LG protests.

Overall, both news articles and user comments expressed relatively low levels of anger (Fig. 5; see Table S7 for distributions). Nevertheless, anger was substantially more prevalent in texts covering LG than in those covering FFF, both in news articles (WMW odds = 0.20, ${\hat{p}}^{* }$(1450) = 23.56, *p* < 0.001) and in user comments (WMW odds = 0.25, ${\hat{p}}^{* }$(151) = 34.23, *p* < 0.001). This confirms that radical protests were accompanied by markedly more anger in media discourse than moderate activism.

We hypothesized that anger expressed in recent LG protest coverage would predict increased support for FFF, consistent with theories linking anger to political engagement and collective action. To test this, we modeled temporal trends in FFF stances as a function of the mean anger expressed in protest-related news coverage over the prior 30 days, the political orientation of outlets, and protest type (LG vs. FFF), using the same counterfactual and modeling framework as in the previous analyses (see Supplementary A4).

In news articles, neither the three-way interaction nor any two-way interactions involving anger were significant (all *p* > .85; Table S8). After model simplification (Table S2), only main effects remained. Contrary to our hypothesis, higher levels of anger in recent coverage predicted *less* supportive stances toward FFF (OR = 0.71, *p* < 0.001), corresponding to a 29% decrease in the odds of more favorable stances per unit increase in anger. Importantly, this effect did not differ between LG and FFF coverage, indicating that anger did not function as a radical flank mechanism in news reporting.

In user comments, the three-way interaction between anger, political orientation, and protest type was again non-significant (OR = 3.67, *p* = 0.08), and no interaction involving anger survived model selection (all *p* > 0.25; Table S8). However, the main effect of anger was positive (OR = 1.12, *p* = 0.03): higher anger in prior news coverage was associated with more supportive stances toward FFF in user comments. As in news articles, this effect did not differ between LG and FFF coverage and therefore does not constitute a RFE.

Taken together, these results indicate that anger is associated with qualitatively different roles in institutional media and user responses—predicting more negative portrayals in news articles but more supportive reactions in user comments, yet does not predict RFEs under either context.

### Target-specific anger analyses (post hoc)

The RFE presupposes that negative reactions toward radical activists may spill over onto evaluations of more moderate groups, either by contrast or contamination. However, anger expressed in protest coverage may be directed at different targets, including governments, corporations, or climate change itself. To assess whether the effects of anger depend on its target, we conducted an exploratory post hoc analysis distinguishing anger directed at climate activists from anger expressed in defense of activists against other entities (see Supplementary Materials, Section D; Box S4; Fig. S9).

Consistent with the theoretical logic of RFEs, we focus primarily on anger attacking climate activists, as only this form plausibly generates contrast or backlash effects between radical and moderate groups. Re-estimating the H3 models using a 30-day rolling mean of activist-directed anger yielded no evidence of an RFE in news articles: neither the three-way interaction nor any lower-order interactions involving anger were significant (all *p* > 0.07; Table S9). In user comments, however, we observed a significant three-way interaction between activist-directed anger, political orientation, and protest group (*OR* = 4.12, *p* = 0.01). Post-hoc comparisons indicated that anger directed at LG predicted more negative trajectories of FFF support relative to the counterfactual baseline implied by FFF coverage. However, this contrast did not survive the Bonferroni–Holm correction for multiple comparisons (Δ*β* = 1.24, *SE* = 0.51, *p* = 0.06) and therefore does not provide robust evidence for an RFE.

For completeness, we also estimated parallel models for anger defending climate activists. Across both news articles and user comments, these models yielded no significant effects of anger, neither as main effects nor within interactions (Table S10). Thus, anger expressed in defense of activists does not appear to shape evaluations of FFF in a way consistent with radical flank dynamics.

Taken together, these target-specific analyses reinforce our main conclusion: even when anger is explicitly directed at climate activists, it does not robustly predict RFEs in either institutional media or user comments.

## Discussion

This study examined how disruptive climate activism reshapes evaluations of more moderate activism in a real-world media environment. Across news articles and user comments, we identify three core patterns. First, short-term exposure to radical protests reliably produces RFEs, but their direction depends on political context: recent coverage of LG increased relative support for FFF in left and right leaning news outlets and left-leaning user comments, while decreasing it in right-leaning comments. Second, these effects are not sustained over longer time periods. Neither the emergence of LG nor cumulative protest coverage produced robust long-term increases in support for FFF, indicating that RFEs are largely transient rather than enduring, with the partial exception of a negative long-term RFE in left-leaning user comments. Third, although anger was more prevalent in coverage of radical protests and correlated with subsequent changes in support for FFF, these effects did not differ from those of the baseline coverage of moderate protests; thus, anger did not specifically predict RFEs. Exploratory analyses suggested that anger directed specifically at activists may actually reduce support for FFF, but this effect was not robust. Taken together, these findings suggest that disruptive protest primarily reshapes short-term evaluative dynamics, often amplifying ideological polarization among news consumers, without generating durable shifts in public support for moderate climate activism.

The most robust finding of this study concerns short-term radical flank dynamics. Monthly coverage of LG protests consistently altered the evaluations of FFF relative to the counterfactual baseline implied by FFF coverage itself, demonstrating that RFEs operate on short time scales. In news articles, recent LG coverage was associated with more favorable shifts of FFF support across political orientations. Crucially, this effect was largely driven by declining support following repeated FFF coverage, rather than by rising support following LG coverage. This suggests that, without a radical flank, fatigue might affect support for climate activism in the news media.

In user comments, short-term effects were strongly influenced by ideology. In left-leaning outlets, increased LG coverage was followed by rising support for FFF, consistent with a positive RFE. In right-leaning outlets, recent LG coverage predicted declining support, indicating a negative RFE. Rather than uniformly enhancing the standing of moderate actors, coverage of disruptive protests thus amplified ideological polarization among readers, increasing support for moderate activism within sympathetic media environment audiences while further alienating oppositional ones.

Previous research on RFEs in climate activism has reported mixed and often inconsistent findings^9,10,26–28^. Few studies have explicitly examined political orientation as a moderator, and those that did yielded heterogeneous results, emphasizing factors, such as prior sympathy for activists rather than ideology per se^9,28^. However, political orientation is closely aligned with baseline sympathy toward climate activism, making it a plausible proxy for such predispositions^30^. From this perspective, our findings suggest that the political leaning of the media outlet functions as a key contextual moderator, helping to reconcile divergent results in the existing literature.

One plausible explanation for this ideological asymmetry lies in baseline evaluations and framing environments. Right-leaning outlets and their audiences evaluated FFF more negatively^20,21,30^, even before LG’s emergence (see Fig. 1), leaving greater scope for backlash once radical tactics entered the media agenda. In this context, LG protests may have reinforced pre-existing skepticism toward climate activism more broadly, contaminating evaluations of both radical and moderate actors. By contrast, left-leaning audiences were more receptive to climate action claims and may have perceived LG as a marginal out-group whose presence made FFF seem more reasonable. Importantly, these effects are confined to short time windows, suggesting that ideological framing conditions shape immediate reactions rather than long-run opinion change.

In contrast to these short-term effects, long-term exposure to radical protests did not produce sustained increases in support for FFF beyond the counterfactual baseline. Neither the emergence of LG nor cumulative protest coverage altered long-term trajectories of FFF evaluations in news articles. In user comments, support for FFF increased over time in left-leaning outlets; however, this increase was significantly weaker following cumulative LG coverage than following baseline cumulative FFF coverage. This pattern constitutes a negative long-term RFE, indicating that prolonged exposure to radical protest coverage dampened the otherwise positive trajectory of support for moderate activism among ideologically sympathetic audiences. Thus, while short-term contrast effects favor moderates, sustained radical visibility may gradually erode their relative advantage. Overall, these findings suggest that RFEs are temporally bounded: initial gains driven by contrast can give way to attenuation or fatigue as exposure accumulates.

Similar temporal decay has been observed in related domains, including the effects of climate strikes on internet search behavior^39^, climate concern^14^, or stock prices^40,41^, where initial responses faded quickly. One explanation is that longer time windows allow additional political, economic, and media dynamics to shape attitudes, diluting the impact of individual protest events^42^. For example, prior work has shown that the immediate negative effects of disruptive protests on attitudes dissipate within months and can even reverse, potentially driven by delayed mechanisms, such as increased awareness or issue salience^43^. In light of our null long-term findings, this suggests that such delayed shifts may emerge only after protest activity subsides and immediate emotional reactions recede.

The divergence between news articles and user comments highlights the importance of distinguishing institutional media framing from participatory audience responses. While news articles reflect editorial norms, professional gatekeeping, and strategic framing, user comments represent a self-selected, politically engaged subset of the audience that actively responds to and contests media narratives. Although such comments cannot be taken as a representative of the broader population, they offer insight into how highly engaged individuals interpret and respond to protest coverage. The stronger ideological polarization observed in comments suggests that RFEs may be especially pronounced among audiences already motivated to engage, amplify, or resist activist messaging, rather than among the public at large.

Contrary to our expectations, anger did not function as a robust mechanism driving RFEs. Although anger was more prevalent in coverage of radical protests and predicted subsequent evaluations of FFF, its effects were not specific to LG and did not reliably differ from the counterfactual baseline implied by FFF coverage. Instead, anger played qualitatively different roles across contexts: it predicted more negative portrayals of FFF in news articles but more supportive reactions in user comments. This divergence likely reflects institutional incentives to deploy emotionally charged language to attract attention and engagement^44,45^, whereas audiences may interpret anger as a signal of moral or personal injustice^33^, which can motivate pro-environmental attitudes and actions^35–37^.

When we restricted the analysis to anger explicitly directed at climate activists, a theoretically more plausible pathway for radical flank dynamics, we observed a directional pattern consistent with a negative RFE, whereby anger directed at LG was followed by more negative trajectories of support for FFF. However, this analysis was exploratory and not preregistered. Moreover, the effect did not survive correction for multiple testing and therefore does not provide robust evidence for anger as an underlying mechanism. Taken together, these findings suggest that while activist-directed anger may plausibly contribute to backlash against moderate actors, its role in generating RFE remains tentative and requires confirmatory testing in future, potentially more controlled, studies.

This study has several limitations. Although it offers high ecological validity by analyzing real-world media discourse, its observational design cannot fully rule out alternative explanations for changes in stances toward FFF, such as broader shifts in climate awareness or concurrent political events. To mitigate this, we explicitly modeled temporal trends and defined RFEs counterfactually using FFF coverage as a baseline, allowing us to isolate effects specific to coverage of LG.

Second, our article scraping may not have captured all protest-related content. Compared to Dablander et al. (2024), we retrieved fewer articles, likely due to stricter inclusion criteria and search-engine constraints; however, the broader time window and lack of systematic outlet-specific bias suggest that this does not threaten internal validity.

Third, user comments reflect a self-selected, politically engaged subset of readers and cannot be generalized to the broader population. Comment activity was also concentrated in a small number of outlets and may include coordinated or automated contributions. Nonetheless, such data provide insight into how highly engaged audiences actively interpret and contest media narratives, complementing institutional news coverage.

Finally, we focused exclusively on anger due to the substantial effort required to validate GPT-based emotion annotations. While anger is theoretically central to protest engagement, other emotions, such as hope, anxiety, and moral elevation, may also shape radical flank dynamics and warrant examination in future research.

In sum, this study provides real-world evidence on how disruptive climate activism reshapes evaluations of more moderate movements within contemporary media ecosystems. We show that RFEs emerge reliably in the short term but are strongly conditioned by political context: recent coverage of LG increased relative support for FFF in institutional media and in user comments at left-leaning outlets, while decreasing it in right-leaning outlets’ comments. These effects did not persist over longer time horizons, indicating that RFEs are transient rather than durable. Crucially, our findings highlight a divergence between institutional media framing and participatory audience responses, with user comments revealing pronounced ideological polarization. Although anger was more prevalent in coverage of radical protests and shaped subsequent evaluations of FFF, it did not robustly mediate RFEs. Taken together, these results suggest that disruptive climate activism primarily alters short-term evaluative dynamics within polarized media environments, underscoring both the strategic potential and the clear limits of protest escalation for generating sustained public support.

## Methods

This study was pre-registered (https://osf.io/f5gza). Deviations from pre-registration are explicitly described below.

### Selection of activist groups

We initially included three climate activist groups that were active in Germany, reached national level media coverage, and protested for broader climate action (in contrast to protesting only for a single topic like coal phase-outs): FFF, LG, and Extinction Rebellion (XR). After reviewing the dataset, we excluded Extinction Rebellion (XR) from the analysis because of its limited coverage in the selected news outlets (*N* = 371), and the group’s earlier protest activities predating FFF, which made it difficult to identify opinion shifts attributable to XR. We classified FFF as a moderate group and LG as a radical group due to their fundamentally different protest strategies. FFF primarily organized authorized demonstrations, whereas LG relied on unannounced road blockades and vandalism of public buildings or artworks, introducing a notably more radical form of climate activism in Germany^5,22^. Importantly, this distinction refers to relative tendencies rather than categorical labels. Individual protests may deviate from these patterns; however, LG’s typical tactics were more confrontational and polarizing than those of FFF^5,20,22^.

### Selection of news outlets and data collection

News outlets were selected based on the following criteria: (1) nationwide coverage, (2) general news content (i.e., not focused on a single topic), (3) a readership exceeding 100,000, and (4) the presence of comment sections. Based on these criteria, six outlets were chosen and categorized by political orientation using classifications from eurotopics.net and ground.news. Left-leaning outlets included Die Tageszeitung (TAZ), Süddeutsche Zeitung (SZ), and Zeit, while Welt, Focus, and Frankfurter Allgemeine Zeitung (FAZ) were classified as right-leaning. Additionally, we included two high-readership outlets that lack comment sections: Spiegel (left-leaning) and Bild (right-leaning), as they represent the most-read sources within their respective political spectrums.

Articles’ URLs were retrieved via the search engine DuckDuckGo using the keywords “letzte Generation”, “letzten Generation”, “Aufstand der letzten Generation”, “FFF”, “Fridays-for-Future”, and “Extinction Rebellion”. We collected all URLs indexed by the search engine between August 2018, marking the start of the FFF movement with Greta Thunberg’s school strikes, and December 2023, the end of our scraping period. For LG-specific articles, we only included content published after January 22, 2022, when the group launched its first street blockade. To avoid confounding factors, we excluded all articles published after October 19, 2023, when Greta Thunberg’s comments on the Palestine conflict sparked significant discussion on FFF unrelated to environmental issues.

Following URL collection, we developed custom web scrapers for each news outlet to extract article content, including metadata (e.g., publication date, title, subtitle), and associated user comments. Scraping was conducted using Python (v 3.10.11) and Selenium (v 4.15.2).

### Scraping procedure

Scraping was conducted between December 28, 2023, and January 6, 2024. We initially collected 6,139 news articles (FFF: 2307; LG: 3,401) and 622,213 user comments (FFF: 177,543; LG: 444,670). After exclusions, the final dataset comprised 2376 news articles (FFF: 859; LG: 1517) and 225,121 user comments (FFF: 70,772; LG: 154,349; see Table 2).

**Table 2:** Quantity of news articles and user comments after data exclusion

Activist Group	Left wing				Right wing				Total
	Spiegel	SZ	TAZ	Zeit	Bild	FAZ	Focus	Welt	
News articles									
FFF	97	105	89	135	51	127	74	181	859
LG	146	204	132	276	135	160	157	307	1517
Total	243	309	221	411	186	287	231	488	2376
User comments									
FFF	-	1	2255	24,290	-	810	1441	41,975	70,772
LG	-	141	5729	58,843	-	6786	14,985	67,865	154,349
Total	-	142	7984	83,133	-	7596	16,426	109,840	225,121

Articles were filtered to include only those containing at least one mention of “FFF”, “Fridays”, “Greta Thunberg,” “Letzte Generation”, “Letzten Generation”, or the commonly used nicknames for LG “Chaoten” and “Kleber”. Articles with only one to three mentions (*N* = 828) were manually screened, and irrelevant items were excluded (*N* = 8). For full exclusion details, see Table S1 in the supplementary materials.

News article distribution was relatively balanced, with most outlets contributing 9–12% of the dataset, except for Bild.de (7.8%), Welt.de (21%), and Zeit.de (17%). Comment data, however, was highly skewed: 86% of all comments came from Welt.de and Zeit.de. For FFF-related articles, these two sources accounted for 93.5% of user comments. As this is one of our primary outcomes, we only included Welt.de and Zeit.de when analyzing commentary data.

### Language processing and model validation

We used GPT-4 Turbo to annotate texts for stances toward FFF and expressed anger, as it outperforms traditional methods for stance detection and word frequencies^46^. Stances were rated on a 5-point scale: 1 = strongly against, 2 = slightly against, 3 = neutral, 4 = slightly in favor, and 5 = strongly in favor. Anger was rated on a 3-point scale: 1 = no anger, 2 = low anger, and 3 = high anger. These were later recoded to -2 to 2 for stances and 0 to 2 for anger. For user comments, we added an “unrelated” label for stance annotations to account for comments that did not directly discuss FFF or had unclear targets. The exact annotation prompts are available in Supplementary Box S1–S2.

To validate GPT’s annotation, we compared its results to the majority ratings of three human raters. After censoring news outlets’ names to reduce bias, human raters annotated a random sample of 160 news articles and 150 user comments on FFF for stance, and 160 articles plus 180 comments on LG for anger. Detailed rating instructions for stance and anger assessments are provided in Supplementary Box S3. No majority agreement was reached in 40 of 310 texts for stances (23 articles, 17 comments) and 42 of 340 for anger (20 articles, 22 comments). These were excluded from the validation procedure.

Inter-rater agreement was measured using Kendall’s W (for ordinal agreement), intraclass correlation coefficients (ICC) for consistency, and Fleiss’ Kappa (K) for nominal data. W is suited for ordinal data and assesses the exact agreement between raters^47^. Consistency ICC assesses the similarity between raters while allowing systematic differences between rating levels^48^. K is used for nominal data and is only reported for the ‘unrelated’ category^47^.

Following validation, all texts were processed by GPT via Microsoft Azure’s GPT API in Python. GPT failed to annotate 6 FFF-related comments and 17 LG-related comments that contained no substantive information and consisted only of a few symbols or brief, non-informative phrases.

### Statistical analysis

All analyses were conducted in R v4.3.2. To account for the ordinal nature of the data, we used proportional odds (PO) regression models. The PO model belongs to the family of cumulative link models, which assume that ordinal outcomes reflect categorized values of an underlying continuous latent variable^49^.

Models for news articles included news outlets as random effects. For user comments, no random effects were included, as only one left-leaning and one right-leaning outlet were analyzed. All models were estimated using the *ordinal* package (v2023.12-4.1) as follows.

Model equation for H1-related PO model

$${{Stance}}_{{FFF}}={LG}\,{{emergence}}_{{Post}}* {Political}\,{{orientation}}_{{right}}* {Time}+1{|Media}$$

Outcome variables are stances towards FFF in each text. *LG emergence* is a binary variable (Pre vs Post) indicating whether disruptive LG protests had already begun (on 22.01.2022) at the time the text was published. *Political orientation* indicates if the media outlet of the given text is oriented left- or right-wing. *Time* is an integer variable that describes the number of months that passed since the emergence of the FFF protests. A separate intercept (random effect *Media*) was estimated for each news outlet to account for the hierarchical structure of our news article data. For user comments, only one news outlet per political orientation was included in the final analyses, so models without random intercepts were calculated.

Model equation for H2.1 related PO model

$${{Stance}}_{{FFF}}={{\rm{\#}}\,{Articles}}_{{t}_{0}:t}* {Political}\,{{orientation}}_{{right}}* {{Type}}_{{Protest}}+{Time}+1{|Media}$$

The predictor ${{\#Articles}}_{{t}_{0}:t}$ refers to the cumulative number of news articles published since the emergence of FFF protests (t_0_). *Type*_*Protest*_ is a binary variable, indicating whether the article count refers to news articles on FFF (moderate) or LG (radical). Other predictors are the same as those defined above for H1.

Model equation for H2.2 related PO model

$${{Stance}}_{{FFF}}={{\rm{\#}}{Articles}}_{t-30:t}* {Political}{{orientation}}_{{right}}* {{Type}}_{{Protest}}+{Time}+1{|Media}$$

The predictor ${{\#Articles}}_{t-30t}$ refers to the number of news articles published in the 30 days prior to each text, calculated as a moving sum. *Type*_*Protest*_ is a binary variable, indicating whether the article count refers to news articles on FFF (moderate) or LG (radical). Other predictors are the same as those defined above for H1.

Model equation for H3 related PO model

$${{Stance}}_{{FFF}}=\,{{Anger}}_{t-30:t}* {Political}\,{{orientation}}_{{right}}* {{Type}}_{{Protest}}+{Time}+1{|Media}$$

${{Anger}}_{t-30:t}$ refers to the mean anger conveyed in news articles published within the 30 days prior to each text. *Type*_*Protest*_ is a binary variable, indicating whether the article count refers to news articles on FFF (moderate) or LG (radical). Other predictors are the same as those defined above for H1.

To avoid misinterpretation of results, non-significant interaction terms were removed stepwise until all remaining interactions were significant or only main effects remained^50^. The final models are shown in Table S2. Post-hoc pairwise comparisons of significant interactions were conducted using the emmeans package (v1.10.5), with p-values adjusted using the Holm-Bonferroni correction.

Like logistic regression for multi-category outcomes, the PO model estimates one intercept per outcome level. However, unlike multinomial models, it requires only a single estimate per predictor, taking advantage of the ordinal structure of the dependent variable^51^. This simplification relies on the PO assumption, stating that the effect of a predictor remains constant across all outcome thresholds^52^.

This assumption can be tested using likelihood-ratio (LR) tests by comparing the PO model to a partial proportional odds (PPO) model that allows predictor effects to vary across levels. A significant LR test suggests a better fit for the PPO model, indicating a violation of the PO assumption^51^. However, in large samples, even minor deviations can yield significant results^53^.

Visual checks can complement these tests by plotting predictor effects across outcome levels^52^, though, to our knowledge, such diagnostics are typically limited to additive models. In cases of violations, Harrell (2015) argues that PO models may still be preferable because they provide easy-to-interpret average effect estimates and are less complex than nominal alternatives. Following this guidance, we tested the PO assumption to avoid misinterpretation but retained PO models when only minor violations were detected. Details on assumption checks and further discussion are provided in Supplementary Materials, section C, Figs. S1–S8.

Brunner-Munzel-tests were used to assess group differences in stance and anger annotations (*brunnermunzel* package v2.0). This test is a more robust alternative to the Mann-Whitney-U test as it relaxes the often-violated assumption of exchangeability^54^. Following Karch (2021), we denote the Brunner-Munzel statistic as ${\hat{p}}^{* }$ and report Wilcoxon-Mann-Whitney (WMW) odds, interpreted similarly to odds ratios.

### Deviations from pre-registration

In our updated pre-registration, we planned to estimate proportional mixed-effects models for all hypotheses. We realized, however, that almost all user comments (93.5%) came only from one left-wing (Zeit) and one right-wing (Welt) news outlet. For this reason, we considered only data from these outlets for the user comments models and calculated models without random effects, since the variable encoding political orientation already accounted for their individual effects. Furthermore, we planned to conduct additional exploratory analyses, controlling for total news coverage of climate change and investigating emotions other than anger. Due to resource limitations, we did not conduct these analyses or collect the respective data. An update to our original pre-registration was conducted at an earlier stage of our study after validating our language models. The exact updates and respective explanations can be found in our pre-registration under https://osf.io/f5gza.

## Supplementary information

Supplementary information

## Data Availability

We worked with copyrighted data, so we cannot publish the news texts themselves. However, we uploaded a dataset containing the date, media, political orientation, support, and anger annotations, as well as our testing set to: https://osf.io/yr7gk.

## Code availability

All analysis scripts are available at https://osf.io/yr7gk.

## References

[1] O'Brien, K., Selboe, E. & Hayward, B. M. Exploring youth activism on climate change: dutiful, disruptive, and dangerous dissent. *Ecol. Soc.* **23**, 26799169 (2018).

[2] Han, H. & Ahn, S. W. Youth mobilization to stop global climate change: narratives and impact. *Sustainability* **12**, 4127 (2020).

[3] Nisbett, N. & Spaiser, V. Moral power of youth activists – transforming international climate politics? *Glob. Environ. Change* **82**, 102717 (2023).

[4] Fisher, D. R. & Nasrin, S. Climate activism and its effects. *WIREs Clim. Change* **12**, e683 (2021).

[5] Kinyon, L., Dolšak, N. & Prakash, A. When, where, and which climate activists have vandalized museums. *npj Clim. Action* **2**, 1–4 (2023).

[6] Feinberg, M., Willer, R. & Kovacheff, C. The activist’s dilemma: extreme protest actions reduce popular support for social movements. *J. Pers. Soc. Psychol.* **119**, 1086–1111 (2020). 31928025 10.1037/pspi0000230

[7] Vandeweerdt, C. The activist’s trade-off: climate disruption buys salience at a cost. (Springer, 2024).

[8] Foxe, J., Dolsak, N. & Prakash, A. Varieties of climate activism: assessing public support for mainstream and unorthodox climate action in the United Kingdom. *Environ. Res. Commun.*10.1088/2515-7620/ad9382 (2024).

[9] Dasch, S., Bellm, M., Shuman, E. & van Zomeren, M. The radical flank: curse or blessing of a social movement? *Glob. Environ. Psychol*. https://psycharchives.org/en/item/7cf21891-1dd0-4c98-9bf2-faf9142dca42 (2023).

[10] Fuller, K. et al. Extreme protest tactics reduce support for the climate movement and climate mitigation policies. Preprint at 10.31235/osf.io/n6aw2 (2025).

[11] Bugden, D. Does climate protest work? Partisanship, protest, and sentiment pools. *Socius* **6**, 2378023120925949 (2020).

[12] Özden, J. & Glover, S. Disruptive climate protests in the UK didn’t lead to a loss of public support for climate policies. https://forum.effectivealtruism.org/posts/YDtsGHmDJMsAWB7Wt/disruptive-climate-protests-in-the-uk-didn-t-lead-to-a-loss (2022).

[13] Kountouris, Y. & Williams, E. Do protests influence environmental attitudes? Evidence from extinction rebellion. *Environ. Res. Commun.* **5**, 011003 (2023).

[14] Brehm, J. & Gruhl, H. Increase in concerns about climate change following climate strikes and civil disobedience in Germany. *Nat. Commun.* **15**, 2916 (2024). 38575557 10.1038/s41467-024-46477-4 PMC10995135

[15] Scheuch, E. G., Ortiz, M., Shreedhar, G. & Thomas-Walters, L. The power of protest in the media: examining portrayals of climate activism in UK news. *Humanit. Soc. Sci. Commun.* **11**, 1–12 (2024).

[16] Wouters, R. From the street to the screen. Characteristics of protest events as determinants of television news coverage. *Mobilization*. **18**, 83–105 (2013).

[17] Chinn, S., Hart, P. S. & Soroka, S. Politicization and polarization in climate change news content, 1985-2017. *Sci. Commun*. https://journals.sagepub.com/eprint/8KYN8ZEWFWKJG5HEIY33/full (2020). 10.1177/1075547020950735 PMC7447862 38602988

[18] Dietze, P. & Craig, M. A. Framing economic inequality and policy as group disadvantages (versus group advantages) spurs support for action. *Nat. Hum. Behav.* **5**, 349–360 (2021). 33318662 10.1038/s41562-020-00988-4

[19] Huang, J., Cook, G. G. & Xie, Y. Large-scale quantitative evidence of media impact on public opinion toward China. *Humanit. Soc. Sci. Commun.* **8**, 1–8 (2021).38617731

[20] Dablander, F., Wimmer, S. & Haslbeck, J. Media coverage of climate activist groups in Germany. *Clim. Change* **178**, 144 (2025). 40718428 10.1007/s10584-025-03959-8 PMC12287173

[21] Meyer, H., Farjam, M., Rauxloh, H. & Brüggemann, M. From disruptive protests to disrupted news frames: comparing German news on climate protests. *Journalism*10.1177/14648849251372805 (2025).

[22] Lederer, M., Lasso Mena, V., Marquardt, J., Richter, T. A. & Schoppek, D. E. Radical climate movements-is the hype about "eco-terrorism" analogy, warning or propaganda?. *Front. Polit. Sci* **6**, 1421523 (2024).

[23] Haines, H. H. Black radicalization and the funding of civil rights: 1957-1970. *Soc. Probl.* **32**, 31–43 (1984).

[24] Haines, H. Radical flank effects. in 10.1002/9780470674871.wbespm174 (2013)

[25] McCammon, H., Bergner, E. & Arch, S. “Are you one of those women?” Within-movement conflict, radical flank effects, and social movement political outcomes*. *Mobilization* **20**, 157–178 (2015).

[26] Baller, C. R. & Bell, S. E. An ecosystem of tactics: bridging the radical and moderate flanks in a pipeline resistance movement. *Energy Res. Soc. Sci.* **117**, 103714 (2024).

[27] Ostarek, M., Simpson, B., Rogers, C. & Ozden, J. Radical climate protests linked to increases in public support for moderate organizations. *Nat. Sustain.* **7**, 1 (2024).

[28] Simpson, B., Willer, R. & Feinberg, M. Radical flanks of social movements can increase support for moderate factions. *PNAS Nexus* **1**, 110 (2022). 10.1093/pnasnexus/pgac110 PMC9896934 36741469

[29] Cuddy, A. J. C., Fiske, S. T. & Glick, P. The BIAS map: behaviors from intergroup affect and stereotypes. *J. Pers. Soc. Psychol.* **92**, 631–648 (2007). 17469949 10.1037/0022-3514.92.4.631

[30] Köhler, J. K. et al. Reasonable or radical? First-order, second-order, and meta-stereotypes of different climate activists among the German public and climate activists. *J. Environ. Psychol.* **104**, 102594 (2025).

[31] Schwartz, S. E. O. et al. Climate change anxiety and mental health: environmental activism as buffer. *Curr. Psychol.*10.1007/s12144-022-02735-6 (2022). 10.1007/s12144-022-02735-6 PMC8883014 35250241

[32] Stanley, S. K., Hogg, T. L., Leviston, Z. & Walker, I. From anger to action: differential impacts of eco-anxiety, eco-depression, and eco-anger on climate action and wellbeing. *J. Clim. Change Health* **1**, 100003 (2021).

[33] Gregersen, T., Andersen, G. & Tvinnereim, E. The strength and content of climate anger. *Glob. Environ. Change* **82**, 102738 (2023).

[34] Gregersen, T., Stanley, S. K., Andersen, G. & Tvinnereim, E. From anger to activism? How drivers of climate anger predict protest intention and support. *Environ. Behav.* **57**, 679–710 (2025).

[35] Jasper, J. M. Constructing indignation: anger dynamics in protest movements. *Emot. Rev.* **6**, 208–213 (2014).

[36] Contreras, A., Blanchard, M. A., Mouguiama-Daouda, C. & Heeren, A. When eco-anger (but not eco-anxiety nor eco-sadness) makes you change! A temporal network approach to the emotional experience of climate change. *J. Anxiety Disord.* **102**, 102822 (2024). 38159371 10.1016/j.janxdis.2023.102822

[37] Stanley, S. K., Hogg, T. L., Leviston, Z. & Walker, I. Anger about climate inaction: the content of eco-anger shapes emotional and behavioral engagement with climate change. Preprint at 10.31234/osf.io/juc67 (2023).

[38] Brosch, T. From individual to collective climate emotions and actions: a review. *Curr. Opin. Behav. Sci.* **61**, 101466 (2025).

[39] Sisco, M. R., Pianta, S., Weber, E. U. & Bosetti, V. Global climate marches sharply raise attention to climate change: analysis of climate search behavior in 46 countries. *J. Environ. Psychol.* **75**, 101596 (2021).

[40] Ramelli, S., Ossola, E. & Rancan, M. Stock price effects of climate activism: evidence from the first global climate strike. *J. Corp. Financ.* **69**, 102018 (2021).

[41] Schuster, M., Bornhöft, S. C., Lueg, R. & Bouzzine, Y. D. Stock price reactions to the climate activism by Fridays for future: The roles of public attention and environmental performance. *J. Environ. Manag.* **344**, 118608 (2023). 10.1016/j.jenvman.2023.118608 37473554

[42] MacKinlay, A. Event studies in economics and finance. *J. Econ. Lit.* **35**, 13–39 (1997).

[43] Ostarek, M., Klein, L., Rogers, C., Ozden, J. & Thomas-Walters, L. Short and long-term effects of disruptive animal rights protest. *Humanit. Soc. Sci. Commun.* **12**, 1110 (2025).

[44] Berger, J. & Milkman, K. L. What makes online content viral? *J. Mark. Res.* **49**, 192–205 (2012).

[45] Xue, J. et al. Twitter discussions and emotions about the COVID-19 Pandemic: machine learning approach. *J. Med. Internet Res.* **22**, e20550 (2020). 33119535 10.2196/20550 PMC7690968

[46] Mayrhofer, L. et al. GPT outperforms BERT and LIWC for stance and anger detection in German news articles and user comments. Preprint at https://osf.io/nq5bw_v1 (2025).

[47] Gisev, N., Bell, J. S. & Chen, T. F. Interrater agreement and interrater reliability: key concepts, approaches, and applications. *Res. Soc. Adm. Pharm.* **9**, 330–338 (2013). 10.1016/j.sapharm.2012.04.004 22695215

[48] Koo, T. K. & Li, M. Y. A guideline of selecting and reporting intraclass correlation coefficients for reliability research. *J. Chiropr. Med.* **15**, 155–163 (2016). 27330520 10.1016/j.jcm.2016.02.012 PMC4913118

[49] Bürkner, P.-C. & Vuorre, M. Ordinal regression models in psychology: a tutorial. *Adv. Methods Pract. Psychol. Sci.* **2**, 77–101 (2019).

[50] Engqvist, L. The mistreatment of covariate interaction terms in linear model analyses of behavioural and evolutionary ecology studies. *Anim. Behav.* **70**, 967–971 (2005).

[51] Fullerton, A. S. & Xu, J. *Ordered Regression Models: Parallel, Partial, and Non-Parallel Alternatives*. (Chapman and Hall/CRC, 2016). 10.1201/b20060.

[52] Harrell, F. E. *Regression Modeling Strategies: With Applications to Linear Models, Logistic and Ordinal Regression, and Survival Analysis*. (Springer, 2015).

[53] Liu, A., He, H., Tu, X. M. & Tang, W. On testing proportional odds assumptions for proportional odds models. *Gen. Psychiatry* **36**, e101048 (2023). 10.1136/gpsych-2023-101048 PMC10410795 37565234

[54] Karch, J. D. Psychologists should use Brunner-Munzel’s instead of Mann-Whitney’s U test as the default nonparametric procedure. *Adv. Methods Pract. Psychol. Sci.* **4**, 2515245921999602 (2021).

