# Supplementary material for: Radical climate protests shaped portrayals of moderate activists and reader attitudes in German news media
Source: NPJ Clim Action. 2026 Jul 27;5(1):68. doi: 10.1038/s44168-026-00361-7 (PMC13407165; doi:10.1038/s44168-026-00361-7)
Supplement: Supplementary file 1 — Supplementary information [file 44168_2026_361_MOESM1_ESM.pdf]

Radical climate protests shaped portrayals of moderate activists and reader attitudes in German news media

Supplementary materials

Lukas Mayrhofer [1]*; Simon Fassnacht [2]; Markus Foramitti [3]; Jana K. Köhler [4]; Boryana Todorova [1]; Claus Lamm [1]; Mauricio Martins [1,5]*

[1] Social, Cognitive and Affective Neuroscience Unit, Faculty of Psychology, University of Vienna, Austria
[2] Max Planck Institute for Biological Cybernetics, Tübingen, Germany
[3] Department of Clinical and Health Psychology, Faculty of Psychology, University of Vienna, Vienna, Austria
[4] Environmental Psychology, Department of Cognition, Emotion, and Methods in Psychology, Faculty of Psychology, University of Vienna, Austria
[5] CICANT, ECATI, Lusófona University, Lisbon, Portugal

* Corresponding authors at: Liebiggasse, 5, 1010 Vienna, Austria. E-mail addresses: mauricio.martins@univie.ac.at (M. Martins), lukas.mayrhofer@univie.ac.at (L. Mayrhofer)

	Page
Table S1: Number and reasoning of excluded news articles	**3**
Box S1. GPT4-Turbo prompts for news articles	4
Box S2. GPT4-Turbo prompts for user comments	5
Box S3. Labelling instructions for human annotators	6
**A) Model specifications**	7
Table S2. Final model specifications by hypothesis and data source.	8
**B) Descriptive Statistics and Model Results**	
Table S3: Distribution of stance annotations by GPT4-Turbo	11
Table S4: Regression results for the H1-related models	12
Table S5: Regression results for the H2.1 models	13
Table S6: Regression results for the H2.2 models	14
Table S7 Distribution of anger annotations by GPT4-Turbo	15
Table S8: Regression results for the H3-related models	16
**C) Model assumption checks**	17
Figure S1: H1 model for news articles.	17
Figure S2: H1 model for user comments.	18
Figure S3: H2.1 model for news articles.	19
Figure S4: H2.1 model for user comments.	20
Figure S5: H2.2 model for news articles.	21
Figure S6: H2.2 model for user comments.	21
Figure S7: H3 model for news articles.	23
Figure S8: H3 model for user comments.	24
**D) Post hoc analysis of anger targets**	25
Box S4. GPT4.1 prompts to detect the target of anger conveyed in texts.	26
Figure S9: Distribution of anger targets allocated by GPT4.1.	27
Figure S10. Post hoc pairwise comparison of the effect of anger toward attacking climate activists on stances toward FFF in user comments.	29
Table S9: Regression results for anger models attacking climate activists.	30
Table S10: Regression results for anger models defending climate activists.	31

Table S1: Number and reasoning of excluded news articles

Step	Reasoning	Articles	Comments	Explanation
1.	Duplicate comments	0	247,979	Comments included multiple times under the same URL.
2.	Error during text extraction	854	22,535	Articles for which no text could be extracted.
3.	LG articles published before their emergence	92	1,848	Articles discussing LG protests before they turned disruptive or false-positive findings.
4.	Empty article string	536	3,347	Empty string for article texts.
5.	Articles published after observation period	558	30,901	Texts published after Greta Thunberg's Palestine tweet.
6.	Premium articles not accessible by us	164	164	Articles behind paywalls that we could not access.
7.	FFF Articles on antisemitism	56	3,836	News articles on FFF containing "Israel" or "Antisemitism" at least three times.
8.	News articles discussing both groups	72	5,787	Articles mentioning both groups at least 3 times.
9.	LG articles that did not mention LG	893	44,771	Articles found for LG without mentioning them.
10.	FFF articles that did not mention FFF	942	62,026	Articles found for FFF without mentioning them.
11.	Articles discussing both groups found in manual screening	23	2,077	Articles mentioning both groups which were not removed in step 9 were manually screened.
12.	Articles not discussing either group found in manual screening	8	978	Articles not mentioning either group at least twice not removed in step 10 or 11 were manually screened.
13.	News articles not in German language	4	4	Text language was checked using the langdetect library version 1.0.9.

Objective:
Label each text according to its expressed support towards Fridays for Future (FFF) or their members, including Greta Thunberg and Luise Neubauer. Additionally, assess the anger conveyed, regardless of the target.

##**Step 1: Label Support Towards FFF (1 to 5)**
5 (Strongly in favor):
- Positive tone that supports FFF's actions, goals, or members.
- Text portrays FFF's protests as helpful, necessary, or having a positive impact.
- Positive remarks clearly outnumber negative ones.
- Examples include texts praising FFF or refuting initial negative views to support FFF.

4 (Slightly in favor):
- Text contains both positive and negative remarks, but positive tone slightly dominates.
- Statements may not be strong, but overall lean in favor of FFF.
- Interviews without criticism or opposition to FFF but not directly praising FFF.

3 (Neutral):
- The text contains no strong opinions for or against FFF.
- It may balance positive and negative remarks equally, without favoring one side.

2 (Slightly against):
- Text contains both positive and negative remarks, but negative tone slightly dominates.
- Statements are more critical of FFF, though not heavily.
- Interviews where opposing views are presented without much criticism towards them.

1 (Strongly against):
- Text contains a negative tone, criticizing FFF's protests, actions, or members.
- Clearly portrays FFF's actions as harmful, unnecessary, or misguided.
- Negative remarks outnumber positive ones.

Step 2: Label Anger (1 to 3)
1 (No anger):
- The text does not convey any frustration, annoyance, or anger.

2 (Little anger):
- Some frustration or annoyance is conveyed.
- Light sarcasm or mild swearing may appear, but not heavily.

3 (High anger):
- Text conveys strong feelings of anger, frustration, or annoyance.
- Heavy use of sarcasm, frustration, or strong language (swearing) is evident.

You may use these labels based on the text content provided. Ensure to classify the support and anger levels independently, even if the text is neutral in tone towards FFF but expresses high levels of anger.
Ensure you output exclusively looks like this: Support=[the label here in the bracket, only the number]; Anger=[the label here in the bracket, only the number]

Box S1. GPT4-Turbo prompts for news articles

Objective:
Label each text according to its expressed support towards Fridays for Future (FFF) or their members, including Greta Thunberg and Luise Neubauer. Additionally, assess the anger conveyed, regardless of the target.

Step 1: Label Support Towards FFF (1 to 5 or Unrelated)
5 (Strongly in favor):
- Positive tone that supports FFF’s actions, goals, or members.
- Text portrays FFF’s protests as helpful, necessary, or having a positive impact.
- Positive remarks clearly outnumber negative ones.
- Examples include texts praising FFF or refuting initial negative views to support FFF.
4 (Slightly in favor):
- Text contains both positive and negative remarks, but positive tone slightly dominates.
- Statements may not be strong, but overall lean in favor of FFF.
- Interviews without criticism or opposition to FFF but not directly praising FFF.
3 (Neutral):
- The text contains no strong opinions for or against FFF.
- It may balance positive and negative remarks equally, without favoring one side.
2 (Slightly against):
- Text contains both positive and negative remarks, but negative tone slightly dominates.
- Statements are more critical of FFF, though not heavily.
- Interviews where opposing views are presented without much criticism towards them.
1 (Strongly against):
- Text contains a negative tone, criticizing FFF’s protests, actions, or members.
- Clearly portrays FFF’s actions as harmful, unnecessary, or misguided.
- Negative remarks outnumber positive ones.

Unrelated:
- The text does not refer to FFF or its members directly or indirectly, or it is unclear.

Step 2: Label Anger (1 to 3)
1 (No anger):
- The text does not convey any frustration, annoyance, or anger.

2 (Little anger):
- Some frustration or annoyance is conveyed.
- Light sarcasm or mild swearing may appear, but not heavily.

3 (High anger):
- Text conveys strong feelings of anger, frustration, or annoyance.
- Heavy use of sarcasm, frustration, or strong language (swearing) is evident.

You may use these labels based on the text content provided. Ensure to classify the support and anger levels independently, even if the text is neutral in tone towards FFF but expresses high levels of anger.
Ensure you output exclusively looks like this: Support=[the label here in the bracket, only the number or Unrelated]; Anger=[the label here in the bracket, only the number]

Box S2. GPT4-Turbo prompts for user comments

Stance labelling - instructions

Please label each text according to its expressed support towards Fridays for Future (FFF) or their members, notably Greta Thunberg and Luise Neubauer, as proper names, nicknames, or pronouns. The opinions expressed in quotes are considered part of the text and are not to be treated in any other way than other parts of the text. If only a minor part of the text is about FFF, only the respective text snippet is to be used to evaluate the support towards the group. The texts can be classified numerically from 1 ('Strongly Against') to 5 ('Strongly In-favor'), depending on the intensity of support (with 5 being the highest support), or as 'Unrelated' to FFF. Below, find the details of each label:

5. Strongly in favour: This label indicates the text has a positive and supportive tone towards FFF. This means that the text generally leads readers to feel that FFF statements and actions, including protests, were helpful to the cause of climate change, rightful, should be continued, had a positive effect on the world, or served a good cause. Sarcastic remarks that sound positive but are not meant in such a way should not get this label. If the text has both positive and negative remarks towards FFF, choose this label if the number of positive remarks clearly exceeds the number of negative remarks. Similarly, this label should be given if an article starts with a negative view of FFF and then proceeds to discuss why that view is invalid while praising FFF, or if a text takes up FFF's reasoning. Interviews should get this label if the interviewee conveys FFF's views or messages without criticism or opposition and praises FFF to at least some extent.

4. Slightly in favour: This label should be given if any of the following conditions are met: 1) while the text can contain both positive and negative remarks about FFF, the tone is slightly more positive than negative; 2) while the statements about FFF are not very strong, they are slightly more positive than negative. Interviews should get this label if the interviewee conveys FFF's views or messages without criticism or opposition, but FFF itself is not directly discussed.

3. Neutral: This label indicates a neutral tone of the text towards FFF. This means that the text either does not convey strong opinions about FFF and/or contains an equal amount and intensity of statements supporting and against FFF.

2. Slightly against: This label should be given if any of the following conditions are met: 1) while the text can contain both positive and negative remarks about FFF, the tone is slightly more negative than positive; 2) while the statements about FFF are not very strong, they are slightly more negative than positive. Interviews should get this label if the interviewee conveys views or messages opposing FFF without criticism or opposition, but FFF itself is not directly discussed.

1. Strongly against: This label indicates a negative tone of the text towards FFF. This means that the text generally leads to the feeling that FFF statements and actions, including protests, were harming the cause of climate change, unlawful, a hindrance to other people, that the protests should be stopped, are unnecessary, or that the protesters don't know what they are doing/saying. If the text has both positive and negative remarks towards FFF, choose this label if the number of negative remarks clearly exceeds the number of positive remarks. Similarly, this label should be given if a text starts with a positive view of FFF and then discusses why this view is invalid while criticizing FFF. Interviews should get this label if

the interviewee conveys views or messages opposing FFF without criticism or opposition, and FFF is directly criticized.
Unrelated: This label is to be used only for commentaries and indicates that the text is clearly unrelated to FFF or that it is unclear to whom the opinion is directed. Remarks regarding climate change can be considered as supportive or opposing FFF, even if they are not mentioned per se, IF common demands or arguments of FFF are discussed.

Anger labelling - instructions:

Please label each text according to the intensity of anger, frustration, or annoyance conveyed in a text, while it does not matter who the anger is directed against (e.g. it could be directed towards activists but also towards views opposing protests, politicians, or companies). Opinions expressed in quotes are considered part of the text. The use of swear words, sarcasm should also be taken as expressions of anger. The texts can be classified numerically from 1 (No anger) to 3 (High anger). Anger is not to be confused with a negative stance, negative support, or any other kinds of negative opinions that are expressed without clear indicators of anger. Below find the details for each label:
1. No anger: This label indicates that no clear feelings of anger, frustration or annoyance are conveyed in the text.
2. Little anger: This label indicates that some feelings of anger, frustration, or annoyance are conveyed in the text. Light swear words and sarcasm might be used but not very extensively.
3. High anger: This label indicates that the text conveys severe feelings of anger, frustration, or annoyance. The text involves heavy uses of swear words and sarcasm.

Box S3. Labelling instructions for human annotators

A) Model specifications

A1) Model equation for H1-related PO model

The following equation was used to investigate H1:

$$Stance_{FFF} = LG\ emergence_{Post} * Political\ orientation_{right} * Time + 1 \mid Media$$

Outcome variables are stances towards FFF in each text. *LG emergence* is a binary variable (Pre vs Post) indicating whether disruptive LG protests had already begun (on 22.01.2022) at the time the text was published. *Political orientation* indicates if the media outlet of the given text is oriented left- or right-wing. *Time* is an integer variable that describes the number of months that passed since the emergence of the FFF protests. A separate intercept (random effect *Media*) was estimated for each news outlet to account for the hierarchical structure of our news article data. For user comments, only one news outlet per political orientation was included in the final analyses, so models without random intercepts were calculated.

A2) Model equation for H2.1 related PO model

The following equation was used to investigate H2.1:

$$Stance_{FFF} = \#Articles_{t_0:\,t} * Political\ orientation_{right} * Type_{Protest} + Time + 1 \mid Media$$

The predictor $\#Articles_{t_0:\,t}$ refers to the cumulative number of news articles published since the emergence of FFF protests (t_0). $Type_{Protest}$ is a binary variable, indicating whether the article count refers to news articles on FFF (moderate) or LG (radical). Other predictors are the same as those defined above for H1.

A3) Model equation for H2.2 related PO model

The following equation was used to investigate H2.2:

$$Stance_{FFF} = \#Articles_{t-30\,:\,t} * Political\ orientation_{right} * Type_{Protest} + Time + 1 \mid Media$$

The predictor $\#Articles_{t-30\,:\,t}$ refers to the number of news articles published in the 30 days prior to each text, calculated as a moving sum. $Type_{Protest}$ is a binary variable, indicating whether the article count refers to news articles on FFF (moderate) or LG (radical). Other predictors are the same as those defined above for H1.

A4) Model equation for H3 related PO model

The following equation was used to investigate H3:

$$Stance_{FFF} = Anger_{t-30\,:\,t} * Political\ orientation_{right} * Type_{Protest} + Time + 1 \mid Media$$

$Anger_{t-30\,:\,t}$ refers to the mean anger conveyed in news articles published within the 30 days prior to each text. $Type_{Protest}$ is a binary variable, indicating whether the article count refers to news articles on FFF (moderate) or LG (radical). Other predictors are the same as those defined above for H1.

Table S2. Final model specifications by hypothesis and data source.

Hypothesis	Source	Preregistered highest-order interaction	Final model change
**H1: LG emergence**	Articles	LG emergence × Political orientation × Time	No interactions removed
	Comments	LG emergence × Political orientation × Time	Three-way interaction removed; retained LG emergence × Political orientation and Political orientation × Time
**H2.1: Long-term LG coverage**	Articles	Cumulative coverage × Political orientation × Protest type	Three-way interaction removed; retained two-way interactions
	Comments	Cumulative coverage × Political orientation × Protest type	No interactions removed
**H2.2: Short-term LG coverage**	Articles	30-day coverage × Political orientation × Protest type	Three-way interaction removed; retained two-way interactions
	Comments	30-day coverage × Political orientation × Protest type	No interactions removed
**H3: Anger in coverage**	Articles	Anger × Political orientation × Protest type	Three-way and two-way interactions removed; retained main effects
	Comments	Anger × Political orientation × Protest type	Three-way interaction removed; retained Anger × Political orientation

Note: For each hypothesis, the table reports the highest-order interaction specified in the preregistered model and whether non-significant higher-order interactions were removed in the final analyses. Model simplification followed a stepwise approach, removing non-significant interaction terms while retaining all lower-order terms to facilitate interpretation. Separate models were estimated for news articles and user comments.

B) Descriptive Statistics and Model Results

Table S3: Distribution of stance annotations by GPT4-Turbo

Stance	Left wing		Right wing	
	n	%	n	%
		News articles		
Strongly In-Favour	202	47	158	36
Slightly In-Favour	149	35	124	29
Neutral	30	7	45	10
Slightly Against	33	8	77	18
Strongly Against	12	3	29	7
Total	426	-	433	-
		User comments		
Strongly In-Favour	1,013	12	654	4
Slightly In-Favour	836	10	593	3
Neutral	1,439	17	1,167	6
Slightly Against	2,253	27	5,366	28
Strongly Against	2,920	34	11,173	59
Total [a]	8,461	-	18,953	-
Unrelated [b]	15,829	65	23,022	55

Distribution of stance labels allocated by GPT4 Turbo, both for news articles and user comments, for both political orientations. Both the total number of texts per label and the share of each label on all texts of each category are presented. Percentages for stance labels in user comments refer only to those comments that did not get the “Unrelated” label. Percentages for the “Unrelated” label refer to the share of all comments of the respective political orientation.

[a] Refers to the number of comments for which GPT could provide a valid stance label.

[b] Refers only to the number of comments that were assigned the “Unrelated” label. Percentages refer to the total number of comments after data exclusion.

Table S4: Regression results for the H1-related models

Predictor	OR	SE	95% - CI		z	p
			LL	UL		
News articles						
LG_{onset}	2.82	3.47	0.25	33.32	0.83	.40
$PolOr_{right}$	0.33	1.49	0.15	0.73	-2.76	.006
Time	0.987	1.01	0.966	1.009	-1.15	.25
$LG_{onset} \times PolOr_{right}$	0.006	7.52	< 0.01	0.33	-2.51	.01
LG_{onset} × Time	0.989	1.03	0.937	1.043	-0.42	.67
$PolOr_{right}$ × Time	1.016	1.02	0.987	1.046	1.07	.28
$LG_{onset} \times PolOr_{right}$ × Time	1.10	1.04	1.01	1.20	2.20	.03
$\sigma^2_{media} = 0.17$, $R^2_{McFadden} = .007$, df = 847						
User comments						
LG_{onset} [a]	1.09	1.10	0.90	1.32	0.90	.37
$PolOr_{right}$ [a]	0.49	1.04	0.45	0.53	-16.3	<.001
Time [a]	1.01	1.00	1.006	1.015	4.34	<.001
$LG_{onset} \times PolOr_{right}$ [a]	0.56	1.13	0.44	0.71	-4.86	<.001
LG_{onset} × Time [b]	1.01	1.00	0.998	1.02	1.65	.10
$PolOr_{right}$ × Time [a]	0.98	1.00	0.975	0.986	-6.87	<.001
$LG_{onset} \times PolOr_{right}$ x Time [c]	0.99	1.01	0.97	1.01	-0.84	.40
$R^2_{McFadden} = .041$, df = 27,405 [a]						

H1 model coefficients (effect of LG protest onset on stances towards FFF) for both news articles and user comments related models. Mixed-effects models were used for news articles with news outlets as random effects, and multiple regression models for user comments, as only one news outlet per political orientation was included. Random effect variance, McFadden's pseudo $R^{2,}$ and degrees of freedom are shown at the bottom of each segment. Insignificant interactions in the full models were removed, leaving only significant interactions.

[a] Effect is derived from a model including two-way interactions between political orientation and LG_{onset} as well as political orientation and time.

[b] Effect is derived from a model including two-way interactions of all three variables, but not the (insignificant) three-way interaction.

[c] Effect is derived from the full model, including the three-way interaction.

Table S5: Regression results for the H2.1 models

Predictor	OR	SE	<		z	p
			LL	UL		
			News articles			
#Articles [a]	0.97	1.02	0.93	1.02	-1.32	.19
$PolOr_{right}$ [a]	0.35	1.43	0.17	0.70	-2.96	.003
Time [a]	1.00	1.00	0.99	1.01	0.64	.52
$Protest_{Radical}$ [a]	1.01	1.11	0.82	1.22	0.05	.96
#Articles × $PolOr_{right}$ [a]	1.06	1.03	1.01	1.12	2.53	.02
#Articles × $Protest_{Radical}$ [b]	1.05	1.04	0.97	1.13	1.25	.21
$PolOr_{right}$ × $Protest_{Radical}$ [b]	1.21	1.21	0.83	1.77	.099	.32
#Articles × $PolOr_{right}$ × $Protest_{Radical}$ [c]	0.97	1.06	0.86	1.09	-0.55	.59
$\sigma^2_{media} = 0.22$, $R^2_{McFadden} = .002$, df = 1708 [a]						
			User comments			
#Articles	1.14	1.01	1.12	1.16	12.67	<.001
$PolOr_{right}$	0.59	1.05	0.54	0.64	-12.08	<.001
Time	0.99	1.00	0.991	0.994	-7.97	<.001
$Protest_{Radical}$	1.47	1.05	1.34	1.61	8.20	<.001
#Articles × $PolOr_{right}$	0.83	1.01	0.81	0.85	-18.44	<.001
#Articles × $Protest_{Radical}$	0.94	1.01	0.92	0.96	-6.29	<.001
$PolOr_{right}$ × $Protest_{Radical}$	0.59	1.05	0.53	0.65	-10.35	<.001
#Articles × $PolOr_{right}$ × $Protest_{Radical}$	1.07	1.01	1.05	1.10	5.89	<.001
$R^2_{McFadden} = .038$, df = 54,816						

H2.1. model coefficients (effect of total news coverage on stances towards FFF) for both news articles and user comments related models. Mixed-effects models were used for news articles with news outlets as random effects, and multiple regression models for user comments, as only one news outlet per political orientation was included. Insignificant interactions were removed until only significant interactions remained. This model was also calculated without time as a predictor due to its high correlation with #Articles. However, results did not change significantly.

[a] Effects are derived from the model, including a significant interaction between #Articles × $PolOr_{right,}$ additive effects of the two, as well as Time and Protest type.

[b] Effects are derived from a model including two-way interactions between #Articles, protest type, and political orientation, but not the (insignificant) three-way interaction.

[c] Effect is derived from the full model, including the three-way interaction.

Table S6: Regression results for the H2.2 models

Predictor	OR	SE	95% - CI		z	p
			LL	UL		
News articles						
$Freq_{Articles}$ [a]	0.84	1.06	0.76	0.93	-3.21	.001
$PolOr_{right}$ [a]	0.43	1.40	0.22	0.82	-2.55	.011
Time [a]	1.00	1.00	0.994	1.006	-0.14	.89
$Protest_{Radical}$ [a]	0.70	1.16	0.52	0.94	-2.40	.017
$Freq_{Articles} \times PolOr_{right}$ [b]	1.04	1.03	0.99	1.09	1.44	0.15
$Freq_{Articles} \times Protest_{Radical}$ [a]	1.20	1.06	1.07	1.34	3.22	.002
$PolOr_{right} \times Protest_{Radical}$ [b]	0.94	1.20	0.66	1.34	-0.35	0.73
$Freq_{Articles} \times PolOr_{right} \times Protest_{Radical}$ [c]	1.20	1.12	0.97	1.49	1.69	.09
$\sigma^2_{media} = 0.21$, $R^2_{McFadden} = .004$, df = 1708 [a]						
User comments						
$Freq_{Articles}$	0.90	1.02	0.87	0.94	-5.19	<.001
$PolOr_{right}$	0.23	1.05	0.21	0.25	-30.93	<.001
Time	0.994	1.00	0.993	0.995	-9.54	<.001
$Protest_{Radical}$	0.76	1.04	0.70	0.83	-6.23	<.001
$Freq_{Articles} \times PolOr_{right}$	1.15	1.02	1.10	1.20	6.44	<.001
$Freq_{Articles} \times Protest_{Radical}$	1.18	1.02	1.13	1.23	8.11	<.001
$PolOr_{right} \times Protest_{Radical}$	1.52	1.06	1.37	1.69	7.78	<.001
$Freq_{Articles} \times PolOr_{right} \times Protest_{Radical}$	0.77	1.02	0.74	0.81	-11.13	<.001
$R^2_{McFadden} = .036$, df = 54,816						

H2.2. model coefficients (effect of news coverage within the prior 30 days on stances towards FFF) for both news articles and user comments related models. Mixed-effects models were used for news articles with news outlets as random effects, and multiple regression models for user comments, as only one news outlet per political orientation was included. Insignificant interactions were removed until only significant interactions remained.

[a] Effects are derived from the model, including an interaction between article frequency x protest type.

[b] Effects are derived from the model, including two-way interactions between article frequency, protest type, and political orientation, but not the (insignificant) three-way interaction.

[c] Effect is derived from the full model, including the three-way interaction.

Table S7 Distribution of anger annotations by GPT4-Turbo

Anger levels	FFF				LG			
	Left-wing		Right-wing		Left-wing		Right-wing	
	n	%	n	%	n	%	n	%
				News Articles				
High Anger	10	2	22	5	53	7	114	15
Little Anger	132	31	172	40	552	73	561	74
No Anger	284	67	239	55	153	20	84	11
Total	426	-	433	-	758	-	759	-
				User comments				
High Anger	2012	8	6,293	15	6925	12	20,053	30
Little Anger	10,037	41	18,744	45	25,219	43	23,965	35
No Anger	12,238	50	16,936	40	26,691	45	23,841	35
Total	24,287	-	41,973	-	58,835	-	67,859	-

Distribution of anger labels allocated by GPT4 Turbo, both for news articles and user comments, for both political orientations and activist groups. Both the total number of texts per label and the share of each label on all texts of each category are presented.

Table S8: Regression results for the H3-related models

Predictor	OR	SE	95% - CI LL	95% - CI UL	z	p
News articles						
$Anger_{t-30}$ [a]	0.716	1.00	0.713	0.72	125.6	<.001
$PolOr_{right}$ [a]	0.430	1.00	0.428	0.432	317.5	<.001
Time [a]	1.004	1.00	0.999	1.010	1.15	.13
$Protest_{Radical}$ [a]	1.15	1.00	1.14	1.16	52.33	<.001
$Anger_{t-30} \times PolOr_{right}$ [c]	1.00	1.54	0.43	2.33	-0.01	.99
$Anger_{t-30} \times Protest_{Radical}$ [b]	1.01	3.28	0.10	10.39	0.01	.99
$PolOr_{right} \times Protest_{Radical}$ [c]	1.06	1.38	0.56	1.99	0.18	.86
$Anger_{t-30} \times PolOr_{right} \times Protest_{Radical}$ [d]	.62	10.93	0.01	67.04	-0.20	.84
$\sigma^2_{media} = 0.24$, $R^2_{McFadden} = .04$, df = 1,104 [a]						
User comments						
$Anger_{t-30}$ [a]	1.12	1.06	1.01	1.25	2.14	.03
$PolOr_{right}$ [a]	0.29	1.03	0.28	0.31	-47.88	<.001
Time [a]	0.994	1.00	0.993	0.996	-7.95	<.001
$Protest_{Radical}$ [a]	1.53	1.06	1.36	1.71	7.31	<.001
$Anger_{t-30} \times PolOr_{right}$ [c]	0.99	1.12	0.80	1.24	-0.07	.95
$Anger_{t-30} \times Protest_{Radical}$ [c]	0.66	1.45	0.31	1.37	-1.13	.26
$PolOr_{right} \times Protest_{Radical}$ [a]	0.36	1.06	0.32	0.41	-16.45	<.001
$Anger_{t-30} \times PolOr_{right} \times Protest_{Radical}$ [d]	3.67	2.05	1.04	17.19	1.74	.08
$R^2_{McFadden} = .049$, df = 32,174						

H3. model coefficients (effect of angry language in news coverage of the prior 30 days on stances towards FFF) for both news articles and user comments related models. Mixed-effects models were used for news articles with news outlets as random effects, and multiple regression models for user comments, as only one news outlet per political orientation was included. Insignificant interactions were removed until only significant interactions remained.

[a] Effects are derived from a model without any interaction terms.

[b] Effects are derived from a model including only the one two-way interaction in question.

[c] Effects are derived from a model including three two-way interactions between all three variables, but not the three-way interaction itself.

[d] Effect is derived from the full model, including the three-way interaction.

C) Model assumption checks

C1) Model assumption check for the H1 PO model for news articles

Assumption tests for the H1 model regarding news articles revealed proportional odds for the LG_{onset} variable (LR(4) = 6.26, p = .18), political orientation (LR(4) = 8.05, p = 0.09), and the three-way interaction (LR(28) = 40.62, p = 0.06) but not time (LR(4) = 10.83, p = 0.03). **Figure. S1** shows that time has a larger effect on extreme than central stances. This could reflect the effects of the COVID-19 pandemic or the interaction of time with the occurrence of climate protests in general, which coincided with the pandemic. Left-wing political orientation also exhibits stronger effects on stances compared to right-wing orientation.

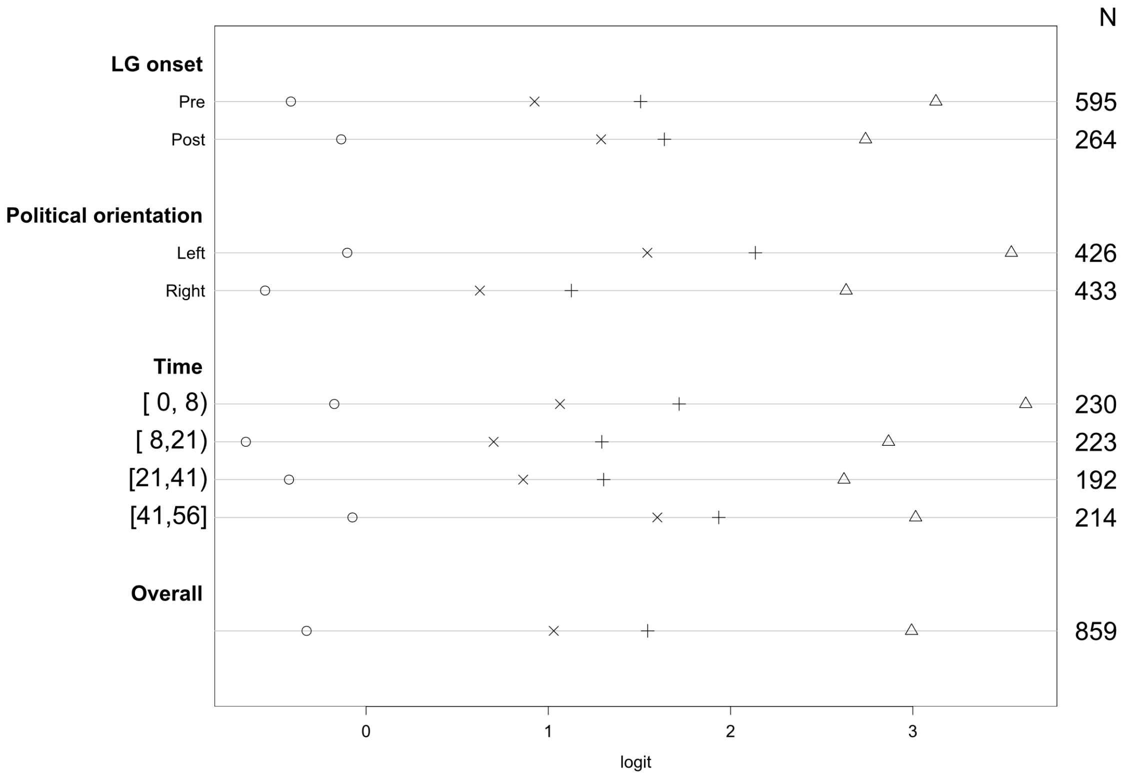

Figure S1. Visual assessment of PO assumption of H1 model for news articles. Logit effects of each predictor for each outcome level. The assumption is met when the effects vertically align on all levels of a predictor for each outcome level. Circular symbols correspond to P(Y = “Strongly in-favor”), x-symbols correspond to P(Y ≥ “Slightly in-favor), plus-signs correspond to P(Y ≥ “Neutral”), and triangles correspond to P(Y ≥ “Slightly against).

C2) Model assumption check for the H1 PO model for user comments

Assumption tests for the H1 model regarding user comments indicated a violation of the PO assumption for all predictors, with $p < .001$ for all likelihood-ratio tests. In a visual inspection, however, additive effects of LG_{onset} and time appear to meet the proportional odds assumption to an acceptable extent, while left-wing political orientation appears to positively interact with outcome levels (**Figure S2**).

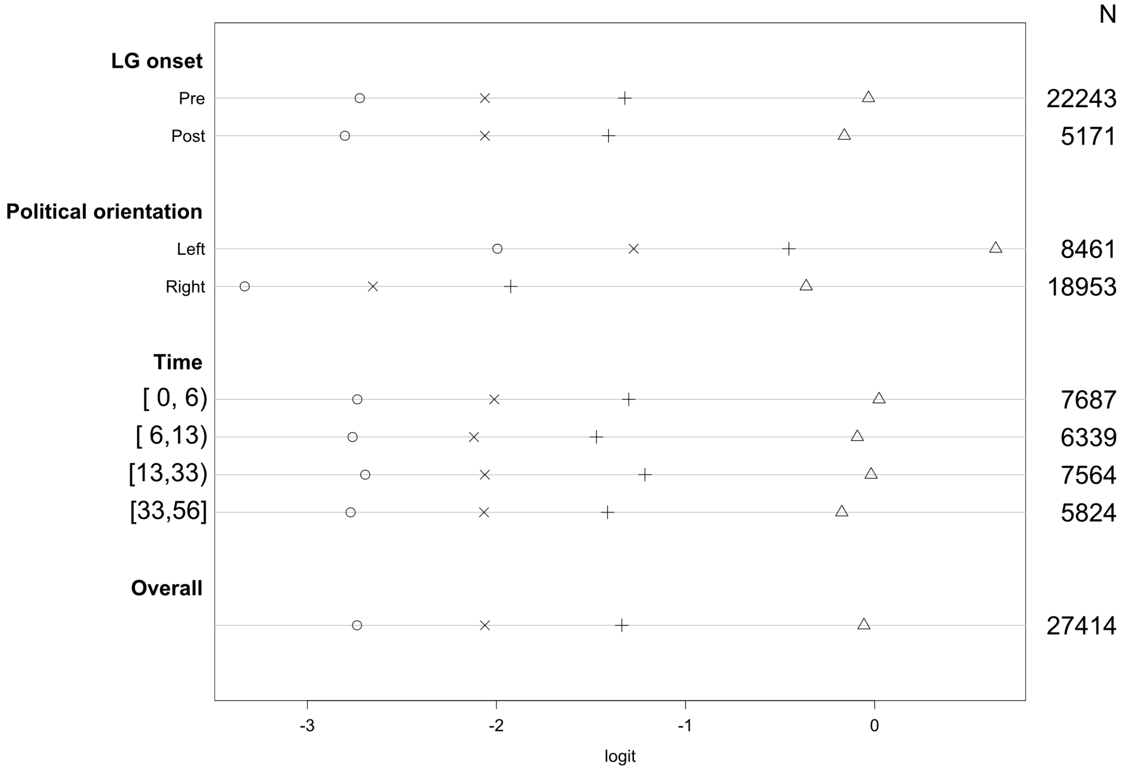

Figure S2. Visual assessment of PO assumption of H1 model for user comments. Logit effects of each predictor for each outcome level. The assumption is met when the effects vertically align on all levels of a predictor for each outcome level. Circular symbols correspond to P(Y = “Strongly in-favor”), x-symbols correspond to P(Y ≥ “Slightly in-favor), plus-signs correspond to P(Y ≥ “Neutral”), and triangles correspond to P(Y ≥ “Slightly against).

C3) Model assumption check for H2.1 PO model for news articles

Assumption tests for the H2.1. models on news articles were significant for time (LR(4) = 21.84, p < .001), political orientation (LR(4) = 15.25, p < .01), the cumulative sum of news articles (LR(4) = 23.79, p < .001) and the two-way interaction (LR(12) = 42.56, p < .001) but not protest type (LR(4) = 0.02, p > .99). In **Figure S3** the cumulative sum of news articles seems to fit the PO assumption adequately, however. Time and political orientation show similar trends to H1, with left-wing orientation and time values near the boundaries associated with larger effects.

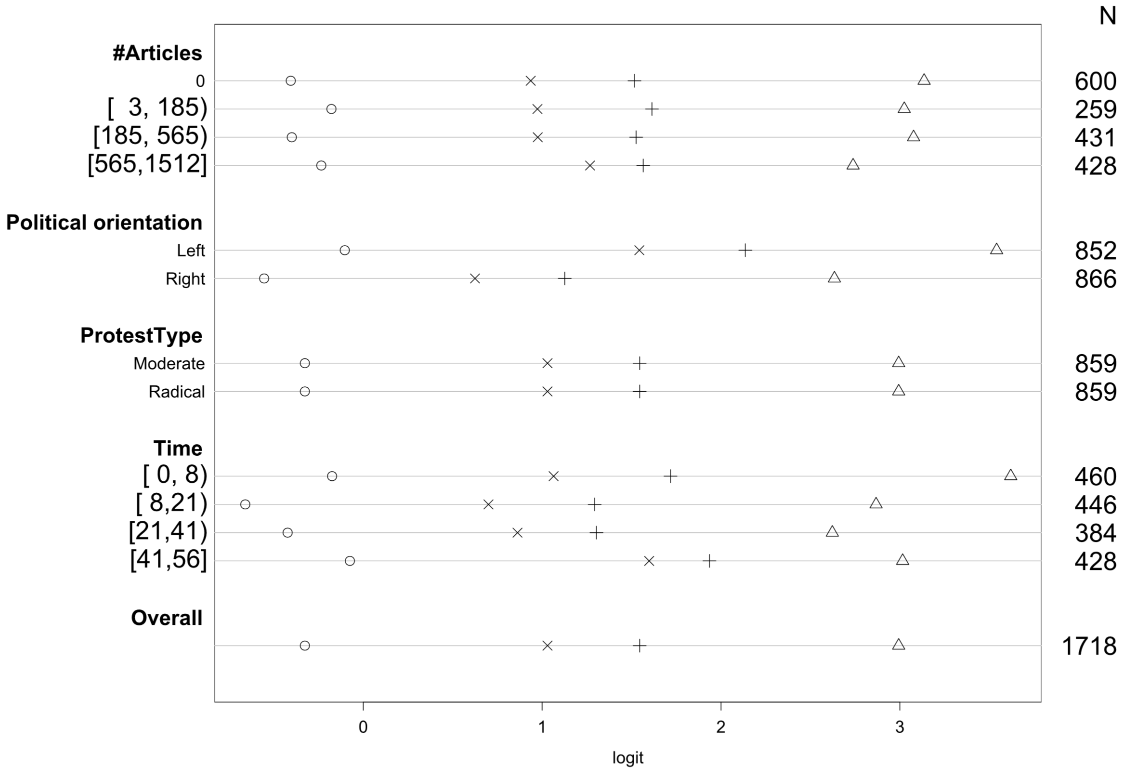

Figure S3. Visual assessment of PO assumption of H2.1 model for news articles. Logit effects of each predictor for each outcome level. The assumption is met when the effects vertically align on all levels of a predictor for each outcome level. Circular symbols correspond to P(Y = “Strongly in-favor”), x-symbols correspond to P(Y ≥ “Slightly in-favor), plus-signs correspond to P(Y ≥ “Neutral”), and triangles correspond to P(Y ≥ “Slightly against).

C4) Model assumption check for H2.1 PO model for user comments

For user comments, significant LR tests are found for all variables with $p < .001$ except for protest type ($LR(3) = 0.01$, $p > .99$). Similar to the assumption tests for H1, **Figure S4** does not provide much evidence for a meaningful assumption violation of the marginal effects except for political orientation.

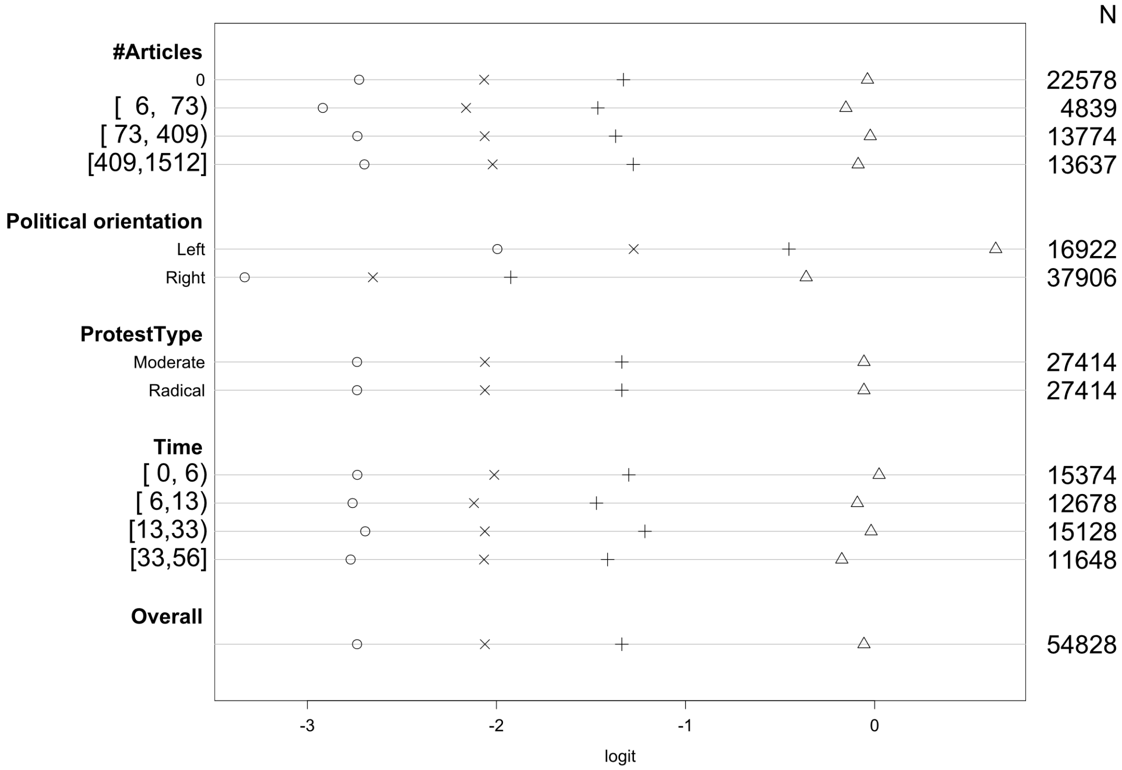

Figure S4. Visual assessment of PO assumption of H2.1 model for user comments. Logit effects of each predictor for each outcome level. The assumption is met when the effects vertically align on all levels of a predictor for each outcome level. Circular symbols correspond to P(Y = “Strongly in-favor”), x-symbols correspond to P(Y ≥ “Slightly in-favor), plus-signs correspond to P(Y ≥ “Neutral”), and triangles correspond to P(Y ≥ “Slightly against).

C5) Model assumption check for H2.2 PO model for news articles

The model investigating effects of article frequency in news articles lead to significant assumption tests for time (LR(4) = 21.91, p < .001) and political orientation (LR(4) = 16.05, p < .01) but not for news article frequency (LR(4) = 8.90, p = .06), protest type (LR(4) = 0.06, p > .99) or the three-way interaction (LR(12) = 16.19, p = 0.18). **Figure S5**, however, shows that the largest deviations occurred for political orientation and time, while the other predictors appeared to meet the assumption.

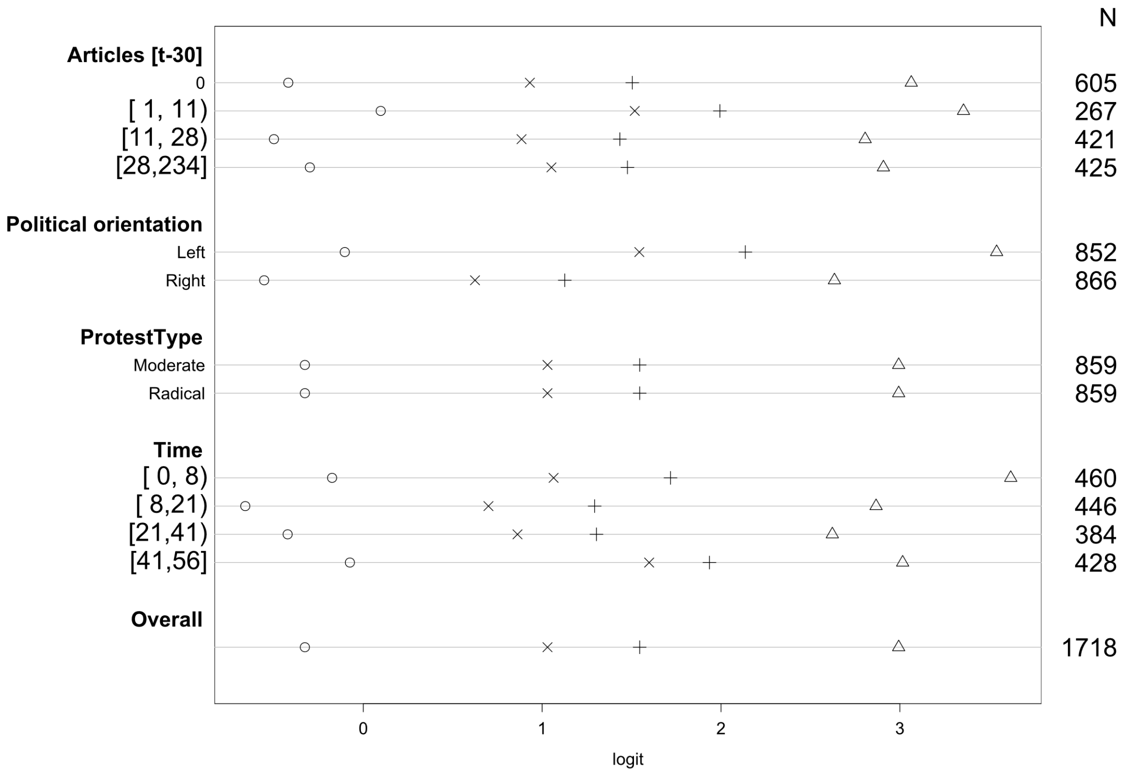

Figure S5. Visual assessment of PO assumption of H2.2 model for news articles. Logit effects of each predictor for each outcome level. The assumption is met when the effects vertically align on all levels of a predictor for each outcome level. Circular symbols correspond to P(Y = "Strongly in-favor"), x-symbols correspond to P(Y ≥ "Slightly in-favor), plus-signs correspond to P(Y ≥ "Neutral"), and triangles correspond to P(Y ≥ "Slightly against).

C6) Model assumption check for H2.2 PO model for user comments

In comments, the effects of time ($LR(3) = 9.73$, $p = .02$), political orientation ($LR(3) = 539.21$, $p < .001$), frequency of articles ($LR(3) = 43.71$, $p < .001$) and the three-way interaction ($LR(21) = 639.63$, $p < .001$) significantly deviated from the proportional odds assumption but not protest type ($LR(3) = 0.24$, $p > .97$). **Figure S6** shows again, that most predictors seem to fit the PO assumption quite well except for political orientation.

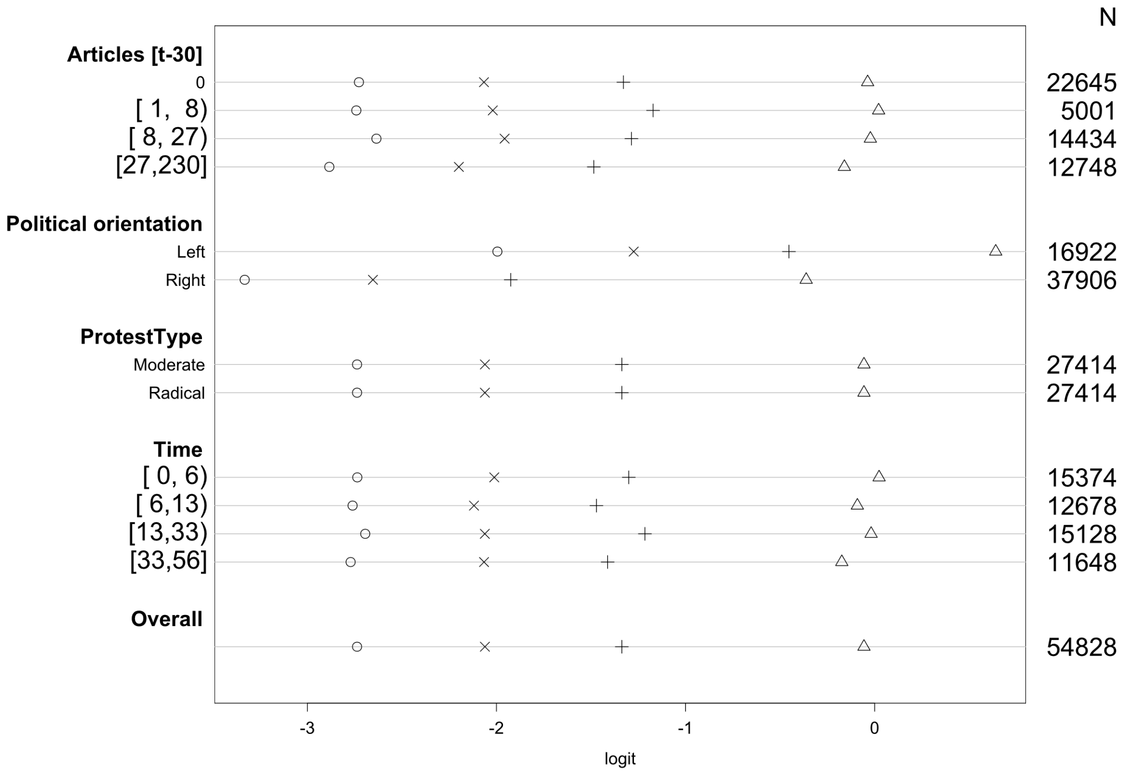

Figure S6. Visual assessment of PO assumption of H2.2 model for user comments. Logit effects of each predictor for each outcome level. The assumption is met when the effects vertically align on all levels of a predictor for each outcome level. Circular symbols correspond to P(Y = "Strongly in-favor"), x-symbols correspond to P(Y ≥ "Slightly in-favor), plus-signs correspond to P(Y ≥ "Neutral"), and triangles correspond to P(Y ≥ "Slightly against).

C7) Model assumption check for the H3 PO model for news articles

Assumption tests in the news article model are significant for time (LR(4) = 15.37, p < .01), political orientation (LR(4) = 13.91, p < .01), and anger (LR(4) = 10.66, p = .03) but not protest type (LR(4) = 4.32, p = .36). -Left-wing political orientation is again associated with stronger effects than right-wing association on all outcome levels (**Figure S7**). Time now shows a pattern different from that in other models, in which longer durations are associated with stronger effects at high outcome levels and weaker effects at low outcome levels. Medium levels of anger, however, seem to be associated with weaker effects at high outcome levels but not at low ones.

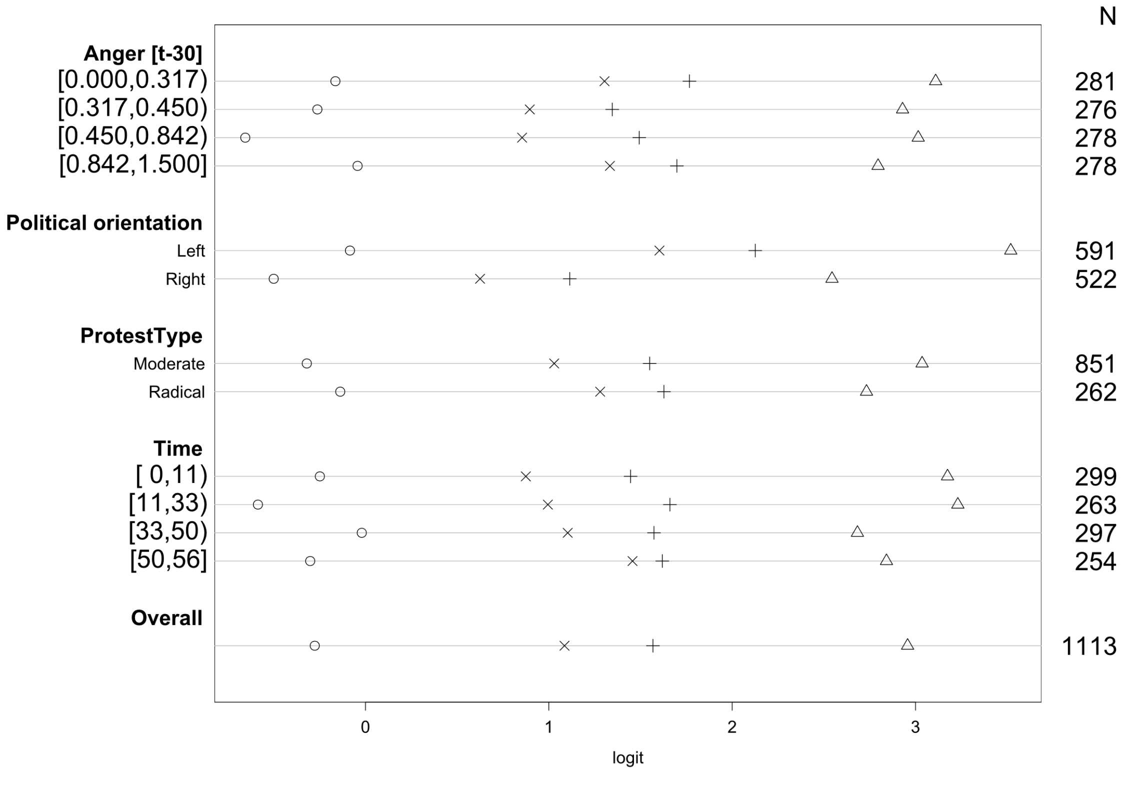

Figure S7. Visual assessment of PO assumption of H3 model for news articles. Logit effects of each predictor for each outcome level. The assumption is met when the effects vertically align on all levels of a predictor for each outcome level. Circular symbols correspond to P(Y = "Strongly in-favor"), x-symbols correspond to P(Y ≥ "Slightly in-favor), plus-signs correspond to P(Y ≥ "Neutral"), and triangles correspond to P(Y ≥ "Slightly against).

C8) Model assumption check for H3 PO model for user comments

In the user comments model, assumption tests were significant for all predictors with $p < .001$ except for time, which was, however, still significant ($LR(3) = 9.96$, $p = .02$). In **Figure S8,** assumption violations do not seem severe except for political orientation.

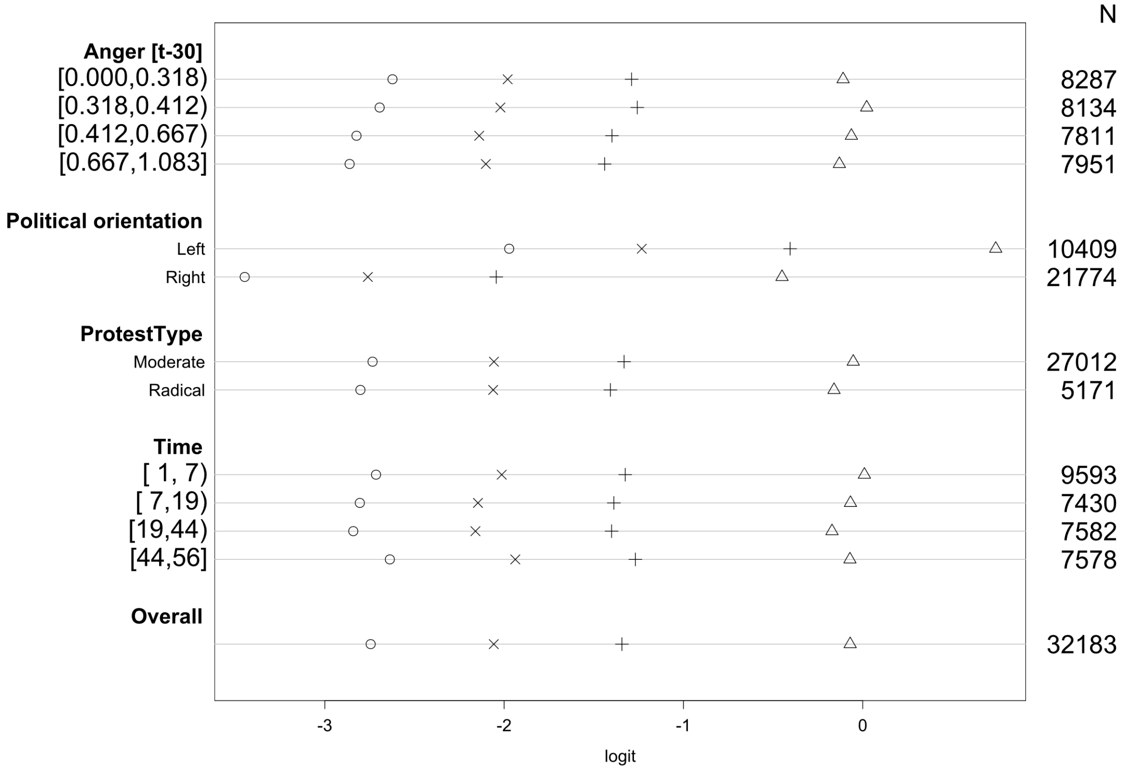

Figure S8. Visual assessment of PO assumption of H3 model for user comments. Logit effects of each predictor for each outcome level. The assumption is met when the effects vertically align on all levels of a predictor for each outcome level. Circular symbols correspond to P(Y = “Strongly in-favor”), x-symbols correspond to P(Y ≥ “Slightly in-favor), plus-signs correspond to P(Y ≥ “Neutral”), and triangles correspond to P(Y ≥ “Slightly against).

D) Post hoc analysis of anger targets

In response to reviewers' concerns about the target of anger, we conducted a non-preregistered post hoc analysis to examine whether anger directed at climate activists moderates the relationship between anger and radical flank effects (H3). This analysis was exploratory and conducted after completion of the preregistered analyses (January 5, 2026).

To identify the target of anger in texts, we developed a new GPT-based prompt (Box S4) that classified anger into four categories: attacking (anger directed at climate activists), defending (anger expressed in defense of climate activists), other (anger directed at unrelated targets), and unclear (ambiguous or mixed targets). Because the exact GPT version used in the preregistered analyses was no longer available, we used GPT-4.1 for this task. The prompt was iteratively refined and validated on a small sample of texts during prompt engineering.

Objective: Label each text according to the target of any anger expressed toward climate activism, in particular the group last Generation or Letzte Generation (LG) and their protest activities. The group is also sometimes referred to as "Klimakleber" or described as climate terrorists or a radical, criminal organization. Climate activist groups that might be targeted by angry language include the last generation which is commonly known for blocking roads or spraying food or paint against government buildings are well known artworks, and Fridays for future which are known for their large-scale climate strikes and demonstrations.

###**Step 1: Label the target of anger**

Attacking: only provide this label when climate activists are explicitly mentioned or referred to and anger is used to attack them. Rules include:
- the anger is expressed to explicitly attack climate activists for their actions or their goals
- the anger conveys frustration caused by governments or media for not acting or speaking out against climate activists and treating them more favorably than their opponents
- the anger conveys frustration caused by the activists
- the text indicates that climate activism is unnecessary and unjustified.

Defending: only provide this label when climate activists are explicitly mentioned or referred to and anger is used to defend them. Rules include:
- the anger is expressed to explicitly defend the climate activists' actions or their goals
- the anger conveys frustration caused by governments, companies, or societies that are responsible for climate change and are ignoring scientists or activists
- the text indicates that climate activism is justified or not as bad as others make it out to be

Other:
- the anger expressed is neither used to defend or attack climate activists

Unclear: provide this label when climate activists are not explicitly mentioned or when it is unclear if anger is used to support or oppose climate activists. Rules include:
- it is unclear whether anger is used to attack or defend climate activists
- the target of anger is ambiguous, and climate activists are not explicitly mentioned as targets of anger
- anger is expressed both to attack and defend climate activists to a similar quantity.

You may use these labels based on the text content provided. Ensure you output exclusively looks like this: Target=[the label here in the bracket, only the string as defined above]

Box S4. GPT4.1 prompts to detect the target of anger conveyed in texts.

Distribution of anger targets

Targets of anger differed markedly across media types, political orientations, and activist groups (Figure. S9). In left-wing news outlets, anger in articles covering FFF was predominantly defensive (73%) and rarely attacking (5%), whereas right-wing outlets showed a more mixed pattern (46% defending, 22% attacking). For LG coverage, left-wing outlets showed similar proportions of defending (27%) and attacking (26%) anger, while right-wing outlets predominantly expressed attacking (49%) anger, with relatively little defensive anger (13%).

User comments exhibited substantially higher levels of anger towards attacking climate activists across both protest groups and political orientations. Compared to news articles, the distinction between anger attacking FFF versus LG was smaller in user comments. In left-wing outlets, comments defended LG slightly more often (23%) than FFF (20%), whereas in right-wing outlets, defensive anger was rare for both groups (FFF: 4%; LG: 1%), and most comments expressed anger attacking activists (FFF: 61%; LG: 75%).

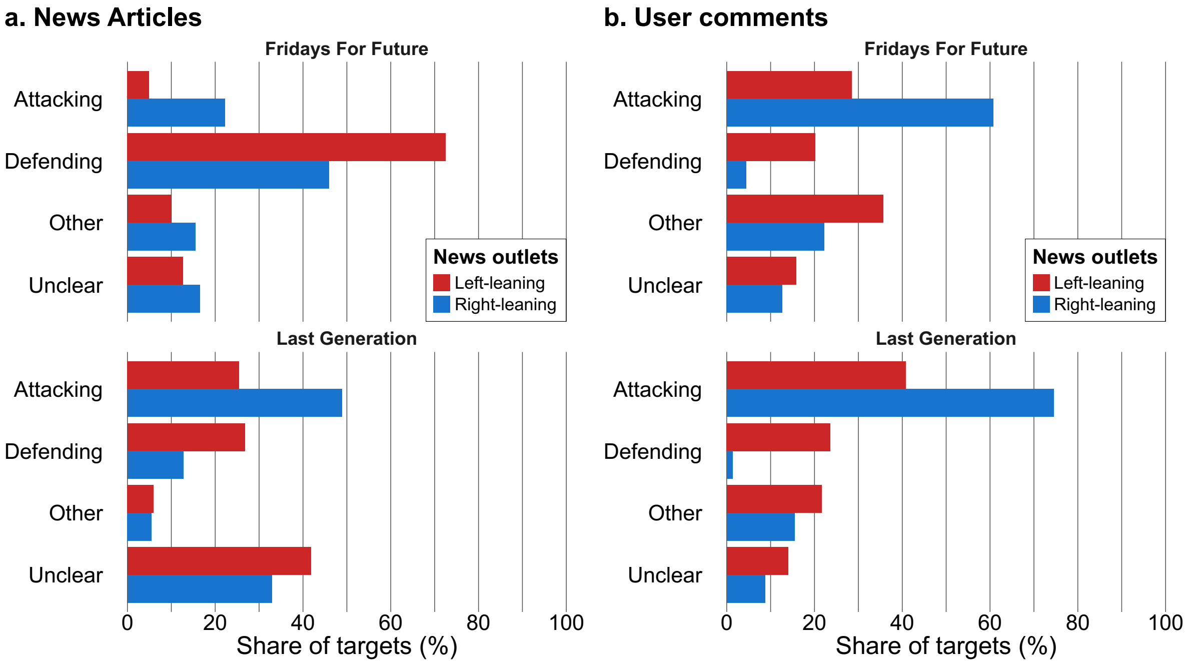

Figure S9. Distribution of anger targets allocated by GPT4.1. Share of anger labels in **a.** news articles and **b.** user comments. Attacking indicates that anger was directed at climate activists. Defending indicates that anger was expressed to defend the activists.

Effects of target-specific anger on FFF stances

To investigate to effect of anger targets, we recomputed the 30-day rolling mean of anger either attacking or defending activists and re-estimated the H3 models using the same specifications as in the preregistered analyses (see Supplementary Methods A4) for both subsets of texts. Texts that were targeted at *Other* entities or in which no targets could be identified (*Unrelated*) were excluded from this analysis.

For news articles, no significant interactions were observed between anger (neither attacking nor defending activists), protest group (LG vs. FFF), and political orientation (Table S9, Table S10). Thus, even when differentiating angry expressions by their targets, there was no evidence that anger predicted radical flank effects in institutional news coverage, consistent with the main analyses.

For user comments, we again found no significant interaction involving anger used to defend activists (Table S10). We did, however, observe a significant three-way interaction between anger attacking activists, protest group, and political orientation ($OR = 4.12$, $p = .01$; Table S9). Post hoc comparisons indicated that, in left-wing outlets, stances toward FFF in user comments became more negative when recent news coverage expressed anger attacking LG compared to the counterfactual baseline implied by FFF coverage (Figure. S10). Importantly, this contrast did not survive Bonferroni–Holm correction for multiple testing ($\Delta\beta = 1.24$, $SE = 0.51$, $p = .06$).

Summary

This post hoc analysis suggests that anger attacking radical activists may be associated with negative spillover effects in user comments under specific ideological conditions. However, these effects were not robust to correction for multiple testing and were not observed in news articles. Consequently, they do not alter the main conclusion that anger - even when considering its targets - does not constitute a reliable or general mechanism underlying radical flank effects in this dataset.

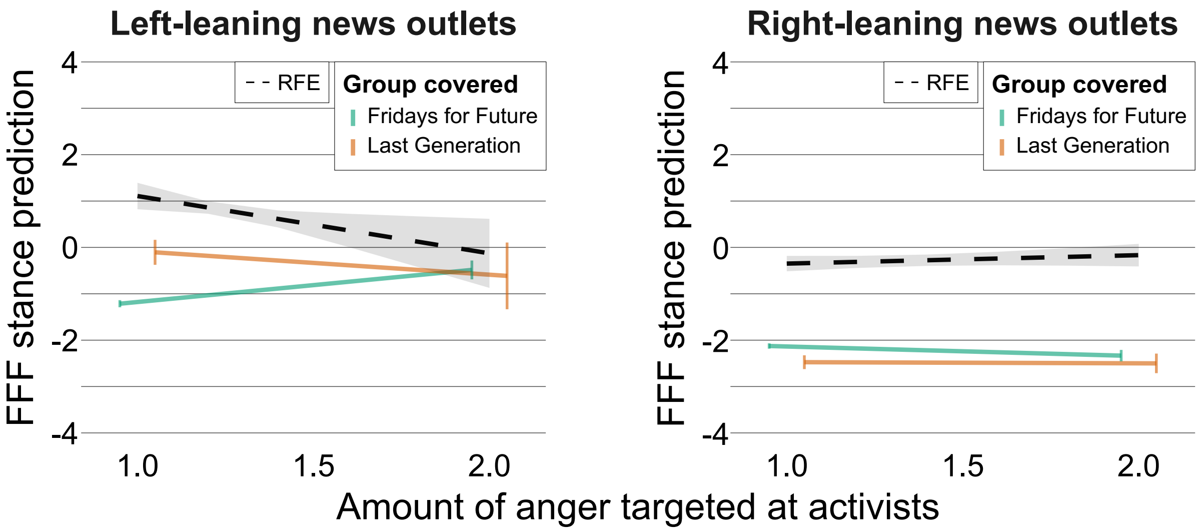

Figure S10. Post hoc pairwise comparison of the effect of anger toward climate activists on stances toward FFF in user comments. The Figureure shows predicted marginal effects with SE bars for the three-way interaction between activist-directed anger in recent news coverage, political orientation of the outlet, and protest group (LG vs. FFF). The dashed line indicates the radical flank effect (LG v FFF), defined as the deviation from the counterfactual baseline implied by FFF coverage. In left-wing outlets, recent news coverage expressing anger toward LG was associated with more negative FFF stance slopes in user comments compared to the counterfactual , although this contrast did not survive correction for multiple testing.

Table S9: Regression results for anger models attacking climate activists.

Predictor	OR	SE	95% - CI		z	p
			LL	UL		
News articles						
$Anger_{t\text{-}30}$ [a]	1.54	.28	.89	2.67	1.55	.12
$PolOr_{right}$ [a]	.43	.38	.21	.91	-2.21	.03
Time [a]	1.00	.01	.99	1.01	.39	.70
$Protest_{Radical}$ [a]	1.10	.21	.73	1.64	.46	.65
$Anger_{t\text{-}30}$ × $PolOr_{right}$ [b]	1.11	.56	.37	3.35	.19	.85
$Anger_{t\text{-}30}$ × $Protest_{Radical}$ [b]	.22	.84	.04	1.16	-1.78	.07
$PolOr_{right}$ × $Protest_{Radical}$ [b]	1.43	.32	.76	2.68	1.11	.27
$Anger_{t\text{-}30}$ × $PolOr_{right}$ × $Protest_{Radical}$ [c]	.62	1.70	.02	17.38	-.28	.78
σ^2_{media} = .24, $R^2_{McFadden}$ = .57, df = 1,104 [a]						
User comments						
$Anger_{t\text{-}30}$ [c]	2.01	.11	1.65	2.59	6.34	>.001
$PolOr_{right}$ [c]	1.02	.15	.76	1.38	.15	.88
Time [c]	.988	.001	.986	.991	-10.24	>.001
$Protest_{Radical}$ [c]	10.42	.64	2.96	36.65	3.65	>.001
$Anger_{t\text{-}30}$ × $PolOr_{right}$ [c]	.39	.13	.30	.51	-7.10	>.001
$Anger_{t\text{-}30}$ × $Protest_{Radical}$ [c]	.29	.51	.11	.78	-2.44	.01
$PolOr_{right}$ × $Protest_{Radical}$ [c]	.06	.68	.02	.21	-4.26	>.001
$Anger_{t\text{-}30}$ × $PolOr_{right}$ × $Protest_{Radical}$ [c]	4.12	.53	1.46	11.71	2.67	.01
$R^2_{McFadden}$ = .40, df = 20710						

H3. model coefficients (effect of angry language in news coverage of the prior 30 days on stances towards FFF) for both news articles and user comments related models. Mixed-effects models were used for news articles with news outlets as random effects, and multiple regression models for user comments, as only one news outlet per political orientation was included. Insignificant interactions were removed until only significant interactions remained.

[a] Effects are derived from a model without any interaction terms.

[b] Effects are derived from a model including three two-way interactions between all three variables, but not the three-way interaction itself.

[c] Effect is derived from the full model, including the three-way interaction.

Table S10: Regression results for anger models defending climate activists.

Predictor	OR	SE	95% - CI		z	p
			LL	UL		
			News articles			
Angert-30 a	1.47	.54	.51	4.23	.71	.48
PolOrright a	.44	.34	.23	.85	-2.43	.01
Time a	1.006	.004	.998	1.013	1.53	.13
ProtestRadical a	.97	.16	.70	1.32	-.22	.83
Angert-30 × PolOrright c	.43	1.13	.05	3.94	-.75	.46
Angert-30 × ProtestRadical c	1.90	1.47	.11	34.01	.43	.66
PolOrright × ProtestRadical c	1.05	.29	.60	1.83	.16	.88
Angert-30 × PolOrright × ProtestRadicald	24.17	2.97	.07	8123	1.07	.28
σ^2_{media} = .19, $R^2_{McFadden}$ = .42, df = 1,104 a						
			User comments			
Angert-30 b	1.02	.08	.86	1.20	.19	.85
PolOrright b	.31	.03	.30	.33	-41.96	>.001
Time b	.995	.001	.993	.996	-6.91	>.001
ProtestRadical b	1.66	.05	1.51	1.84	10.06	>.001
Angert-30 × PolOrright c	1.24	.38	.59	2.59	.57	.57
Angert-30 × ProtestRadical c	1.24	.39	.58	2.66	.56	.58
PolOrright × ProtestRadical b	.34	.06	.30	.38	-17.25	>.001
Angert-30 × PolOrright × ProtestRadicald	2.00	.78	.44	9.13	.89	.37
$R^2_{McFadden}$ = .14, df = 29140						

H3. model coefficients (effect of angry language in news coverage of prior 30 days on stances towards FFF) for both news articles and user comments related models. Mixed effects models are used for news articles with news outlets as random effects and multiple regression models for user comments as only one news outlet of each political orientation was included. Insignificant interactions were removed until only significant interactions remained.

[a] Effects are derived from a model without any interaction terms.

[b] Effects are derived from a model including only the one two-way interaction in question.

[c] Effects are derived from a model including three two-way interactions between all three variables but not the three-way interaction itself.

[d] Effect is derived from the full model including the three-way interaction.
